# CAST-SecOC: A Configuration-Aware Multidimensional Testing Method for AUTOSAR SecOC over CAN FD

**DOI:** 10.3390/s26196004

**Published:** 2026-09-22

**Authors:** Yaozong Xu, Cong Wang, Maode Ma

**Affiliations:** 1 School of Mechanical Engineering, Tianjin University, Tianjin 300072, China; xuyaozong@tju.edu.cn; 2 College of Artificial Intelligence, Tianjin University of Science and Technology, Tianjin 300457, China; wangcongjcdd@tust.edu.cn; 3 Faculty of Computer Science and Artificial Intelligence, Shenzhen University of Advanced Technology, Shenzhen 518107, China

**Keywords:** AUTOSAR SecOC, CAN FD, configuration-aware security testing, multidimensional requirement oracle, freshness-state testing, application-delivery gating, configuration mutation testing, traceable test evidence

## Abstract

AUTOSAR Secure Onboard Communication (SecOC) protects in-vehicle messages through authentication, freshness verification, and application-delivery gating, but deployment security also depends on configuration and receiver-state handling. This paper presents CAST-SecOC, a configuration-aware multidimensional testing method for SecOC over Controller Area Network with Flexible Data-Rate (CAN FD). The method jointly models message semantics, observable receiver conditions, freshness relations, configuration parameters, perturbation operators, requirement-level oracles, and traceable evidence. A prototype campaign produced 1575 execution records from 315 retained semantic equivalence classes. None of the 1249 invalid messages reached the application, while all 286 valid messages were eventually delivered; under high load, six of 126 valid messages exceeded their deadlines. Re-scoring the same message-level records with an application-only binary oracle reduced the outcomes to 286 ACCEPT and 1249 REJECT, whereas CAST-SecOC retained six distinct response/timing signatures. Systematic configuration mutation killed 16 of 20 executable non-equivalent mutants, yielding a mutation score of 80.00%. Composed timing-budget analysis showed that Protocol Data Unit PDU-C and PDU-D retained positive headroom at 30% load but insufficient headroom at 85% and 95% load for all three evaluated authenticated-input lengths. The results show that CAST-SecOC provides executable and mechanism-aware evidence for SecOC deployment verification and configuration-fault analysis.

## 1. Introduction

### 1.1. Background

Automotive electrical and electronic architectures are moving from distributed control toward domain-centralized and central-computing designs. Vehicle control units, battery-management systems, motor controllers, and braking, steering, and body controllers continuously exchange state variables, estimates, and control commands over CAN or Controller Area Network with Flexible Data-Rate (CAN FD). These messages are periodic and subject to explicit end-to-end deadlines. Their integrity, source authenticity, and temporal freshness directly affect the control inputs consumed by the receiving Electronic Control Unit (ECU). Conventional CAN provides neither native message authentication nor replay protection. An adversary with access to the in-vehicle network can therefore eavesdrop on, forge, modify, or replay messages and can increase communication and processing load through burst injection [1,2,3].

AUTOSAR Secure Onboard Communication (SecOC) provides authenticity, integrity, and freshness protection at the Protocol Data Unit (PDU) layer. The sender computes an authenticator from the Authentic I-PDU, Data Identifier (DataID), and full freshness value. The receiver reconstructs the verification freshness value from local freshness information and forwards the message to the application only after authentication, freshness checking, and delivery gating have all succeeded. Production configurations commonly transmit truncated freshness fields and truncated authenticators and may authenticate only a selected region of the Authentic I-PDU. SecOC deployment testing must therefore cover cryptographic computation, authenticated-region selection, freshness reconstruction, acceptance windows, rollover and resynchronization policies, authenticator length, and cryptographic-context mapping [4,5].

CAST-SecOC addresses this deployment-verification chain. It generates test vectors subject to configuration and observable protocol conditions, treats application delivery as the security outcome, and records authentication, freshness, resynchronization, and state updates as the mechanism path. VectorID, ConfigID, bus traces, ECU logs, verdicts, and defect labels are retained in a traceable evidence chain. The resulting diagnosis goes beyond whether a message was rejected: it identifies the blocking mechanism, determines whether receiver state was updated correctly, and links anomalous behavior to the responsible configuration.

From a sensing-system perspective, the messages considered in this study form part of the vehicle sensing–decision–actuation chain. PDU-A carries battery-pack voltage, current, and state-of-charge information; PDU-B carries vehicle and wheel-speed measurements; PDU-C carries motor-speed and actual-torque feedback; PDU-D carries commanded torque and drive-enable information; and PDU-E carries cell-temperature and insulation-status information. For these sensor and actuator PDUs, communication integrity includes not only message authenticity but also freshness, correct security-context binding, and timely delivery. A stale, misbound, or incorrectly released PDU can therefore affect the state information or control command consumed by the receiving ECU. SecOC deployment verification is consequently treated here as part of preserving the integrity of the automotive sensing and control chain.

### 1.2. Related Work

Research on in-vehicle message authentication has largely focused on lightweight protocols, security strength, and schedulability. CANAuth, LiBrA-CAN, vatiCAN, LeiA, S2-CAN, CANASTA, and CAN-MM improve CAN protection through different authentication constructions, deployment strategies, and timing models [6,7,8,9,10,11,12]. These studies focus primarily on authentication-protocol design; the present work instead examines the configuration and receiver behavior of a deployed SecOC link.

Recent studies have extended CAN-FD protection from isolated message authentication to integrated security architectures, key-management mechanisms, and timing-aware deployment. TrinitySec combines authentication, authorization, confidentiality, and integrity protection for CAN-FD communication, whereas DBCSec uses the communication relationships encoded in CAN database (DBC) files to organize key management and secure message exchange [13,14]. Other studies have investigated frame-level encryption and authentication for CAN FD and the joint treatment of communication security and end-to-end latency [15,16]. These studies primarily focus on the design and performance of protection mechanisms, whereas CAST-SecOC examines whether deployed SecOC mechanisms and configuration policies behave in accordance with their requirements.

Studies of SecOC and freshness management have shown that truncated counters, synchronization policies, and resynchronization procedures alter replay-protection boundaries [17,18]. Engineering studies have also demonstrated the feasibility of SecOC implementations, CANoe-based simulation, and adaptation to Chinese commercial cryptographic algorithms [19,20,21]. Building on this work, CAST-SecOC places the authenticated region, freshness relations, rollover gating, cryptographic contexts, and application delivery within one test model and uses a requirement-level oracle to distinguish protocol paths.

Recent protocol-level assessments further show that the security of an in-vehicle network cannot be inferred solely from the presence of a cryptographic primitive. Authentication coverage, replay protection, implementation behavior, protocol composition, and configuration assumptions must be examined together [22,23]. Formal analysis of an AUTOSAR-based security module has also demonstrated the value of verifying security properties at the protocol and software-module levels [24]. CAST-SecOC complements such analysis with execution-based evidence collected from configuration-constrained test vectors and observable receiver responses.

Automotive cybersecurity testing encompasses fuzzing, model-based testing, hardware-in-the-loop testing, and automated test platforms. Prior surveys emphasize that protocol state, input constraints, and feedback quality directly affect test effectiveness [25,26,27,28,29]. CAST-SecOC introduces SecOC-specific constraints and evidence structures: an input combines a message, configuration, freshness relation, and perturbation operator; feedback covers authentication, freshness, application delivery, resynchronization, state updates, and latency; and the metrics separately characterize message security, real-time availability, policy enforcement, and systematic configuration-mutation adequacy.

To further clarify the differences from recent studies, Table 1 compares five representative works in terms of their main objectives, evaluation scale, receiver-state modeling, configuration-boundary coverage, and limitations for SecOC deployment verification. State-condition coverage refers to the two receiver conditions used in CAST-SecOC, SYNCED and ROLLOVER_GUARD, while configuration-boundary coverage follows the nine configuration dimensions defined in Equation (16).

As shown in Table 1, recent studies have mainly focused on authentication design, secure CAN-FD communication, MAC transmission, key management, or schedulability analysis. These works provide useful security and performance evaluations, but they generally do not model receiver conditions and SecOC configuration boundaries together.

CAST-SecOC extends the evaluation from protection-mechanism design to deployment verification. It covers the two receiver conditions and the nine configuration dimensions defined in this work, and it links them with executable test vectors, freshness relations, requirement-level verdicts, and configuration-fault evidence. This enables faults related to freshness handling, rollover gating, authenticated regions, and cryptographic-context mapping to be examined within the same testing framework.

Table 1 is a qualitative comparison of the capabilities reported in the respective studies rather than a head-to-head experimental benchmark. To directly evaluate the diagnostic contribution of the CAST-SecOC oracle, Section 5.5.3 re-scores the same message-level evidence records using an application-only binary accept/reject oracle. This ablation keeps the test inputs and execution evidence unchanged and varies only the information used by the oracle.

### 1.3. Research Questions and Contributions

This study addresses the following research questions:

- RQ1: Can joint modeling of message semantics, observable receiver conditions, freshness relations, configuration parameters, and perturbation operators produce executable and evidence-traceable SecOC test vectors?
- RQ2: Compared with an application-only binary accept/reject oracle, can the multidimensional oracle distinguish defensive mechanism paths, timing violations, and configuration-relevant failures that would otherwise be merged into the same final outcome?
- RQ3: How do authenticated-input length and CAN-FD traffic conditions affect target-platform cryptographic latency and message-level timing, respectively, and what timing headroom remains under the evaluated configurations?

The main contributions are as follows:

(1) A configuration-aware multidimensional test model for SecOC deployment verification. The model represents message semantics, observable receiver conditions, freshness relations, configuration parameters, perturbation operators, condition setup, requirement oracles, and evidence records, while explicitly separating bus-level adversary capabilities from laboratory-only control capabilities.
(2) A constrained method for constructing and prioritizing test vectors. Observable conditions, freshness relations, configuration boundaries, perturbation operators, and security requirements are treated as coverage obligations. Feasibility filtering removes combinations that violate protocol semantics, equivalent-vector reduction eliminates redundant inputs, and coverage gain determines a reproducible execution order under a fixed tie-breaking rule.
(3) A multidimensional oracle that combines the security outcome with the mechanism path. Application delivery determines whether an anomalous message crossed the security boundary, whereas authentication, freshness, resynchronization, and state updates confirm the expected defensive path. Invalid-Acceptance Rate (IAR) and False-Rejection Rate (FRR) quantify message-level security, Deadline-Miss Rate (DMR) quantifies real-time availability, Inconclusive Rate (IR) captures evidence completeness, Number of Accepted Policy-Violation (NPV) records accepted non-compliant configurations, and $MS_{\mathrm{cfg}}$ evaluates configuration-test adequacy using systematically generated mutants derived from the nine configuration dimensions.
(4) A prototype evaluation comprising 1575 execution records from 315 retained semantic equivalence classes, systematic configuration mutation, and target-platform timing measurements. All 1249 invalid messages were blocked, while systematic mutation testing killed 16 of 20 executable non-equivalent mutants (${\mathrm{MS}}_{\mathrm{cfg}}=80.00\%$), with four survivors exposing residual test-suite limitations.

## 2. Technical Background

### 2.1. SecOC Protection Chain and On-Wire PDU

The system considered in this work carries AUTOSAR SecOC traffic over CAN FD. The sender receives an Authentic I-PDU, obtains a full freshness value from the Freshness Manager, and computes the authenticator using the cryptographic context assigned to the message. The receiver parses the Secured I-PDU, reconstructs the full freshness value, and recomputes the authenticator. The Authentic I-PDU is passed upward only when authentication, freshness checking, and application-delivery gating satisfy the applicable requirements.

For message $m_{i}$, the on-wire Secured I-PDU is abstracted as

(1) $$P_{\mathrm{wire},i}=\left[H_{\sec,i}\right]\|P_{\mathrm{auth},i}\left\|\left[FV_{\mathrm{tr},i}\right]\right\|T_{\mathrm{tr},i},$$

where square brackets denote optional fields, $P_{\mathrm{auth},i}$ is the Authentic I-PDU, $FV_{\mathrm{tr},i}$ is the truncated freshness field, and $T_{\mathrm{tr},i}$ is the truncated authenticator. VectorID, ConfigID, state labels, and coverage labels remain at the test controller and are not transmitted on the bus.

A generic KeyIndex is not treated as a standard on-wire field of the Secured I-PDU. Authentication-service references, cryptographic jobs, and key references are internal ECU configuration items. If a project defines an additional on-wire key-selection field, its position, encoding, and inclusion in the authenticated region must be stated explicitly.

### 2.2. Authenticated Region and SM4-CMAC Model

A SecOC implementation may authenticate only a selected region of the Authentic I-PDU. For message $m_{i}$, let

(2) $$A_{i}=\left(o_{i},n_{i}\right)$$

denote the authenticated-region parameters, where $o_{i}$ is the starting offset and $n_{i}$ is the region length. The corresponding set of authenticated byte positions is

(3) $$I_{i}=\left\{o_{i},o_{i}+1,\ldots ,o_{i}+n_{i}-1\right\}.$$

The authenticated input is

(4) $$D_{\mathrm{auth},i}={\mathrm{BE}}_{16}\left({\mathrm{DataID}}_{i}\right)\left\|P_{\mathrm{auth},i}\left[o_{i}:o_{i}+n_{i}-1\right]\right\|{\mathrm{BE}}_{b_{i}}\left(FV_{\mathrm{full},i}\right),$$

where ${\mathrm{BE}}_{k}\left(\cdot \right)$ encodes a value as a *k*-bit field using the project-defined byte order.

The evaluation uses SM4-based Cipher-based Message Authentication Code (SM4-CMAC) as the project-level authentication implementation. This choice serves to evaluate the test method and is not presented as an AUTOSAR restriction on the cryptographic algorithm. The transmitted authenticator is

(5) $$T_{\mathrm{tr},i}={\mathrm{Trunc}}_{\ell_{i}}\left({\mathrm{CMAC}}_{K_{\kappa_{i}}}\left(D_{\mathrm{auth},i}\right)\right),$$

where $\ell_{i}$ is the transmitted authenticator length, $\kappa_{i}$ is the internal cryptographic context assigned to the message, and $K_{\kappa_{i}}$ is the authentication key referenced by that context. Truncation direction, bit order, byte order, and padding are part of the configuration under test and must be recorded with the experiment.

### 2.3. Counter-Based Freshness Reconstruction and Observable Receiver Conditions

The experiments use a counter-based freshness configuration. Let *b* be the width of the full freshness value, λ the transmitted-field length, $x=FV_{\mathrm{tr}}$ the received truncated value, and $FV_{\mathrm{last}}$ the most recently accepted full value. The current high-order estimate is

(6) $$h=\left\lfloor \frac{FV_{\mathrm{last}}}{2^{\lambda }}\right\rfloor .$$

The receiver constructs candidates from the adjacent high-order intervals:

(7) $$C_{FV}\left(x\right)=\left\{\left(h+j\right)2^{\lambda }+x\;|\;j\in \left\{-1,0,1\right\},0\leq \left(h+j\right)2^{\lambda }+x<2^{b}\right\}.$$

Outside a rollover transition, the candidates admissible under the acceptance window are

(8) $$A_{FV}\left(x\right)=\left\{y\in C_{FV}\left(x\right)\;|\;0<y-FV_{\mathrm{last}}\leq W\right\}.$$

The reconstructed value is

(9) $$\hat{FV}=\left\{\begin{array}{cc} \min A_{FV}\left(x\right), & A_{FV}\left(x\right)\neq \emptyset ,\\ ⊥, & A_{FV}\left(x\right)=\emptyset . \end{array}\right.$$

Equations (6)–(9) describe the counter-based reconstruction logic used in the experiments. For composite counters, timestamps, or other candidate-selection rules, the test model can invoke the project-specific rules of the Freshness Manager under test.

Only receiver conditions that can be established independently through the available observation chain are used as state dimensions. The observable-condition set is

(10) $$S_{O}=\left\{\mathrm{SYNCED},\mathrm{ROLLOVER}\text{\_}\mathrm{GUARD}\right\},$$

where SYNCED denotes continuous verification and delivery under stable operation, and ROLLOVER_GUARD denotes the delivery-gated interval after a low-value rollover candidate appears and before an authenticated cycle transition has been confirmed. Loss of synchronization, out-of-window inputs, and historical inputs are represented as freshness relations; resynchronization requests and their authentication basis are represented as response actions. This separation keeps stable operating conditions, input relations, and recovery actions distinct.

The freshness-relation set is

(11) $$R_{FV}=\left\{\mathrm{NEXT},\mathrm{STALE},\mathrm{OUT}\text{\_}\mathrm{OF}\text{\_}\mathrm{WINDOW},\mathrm{WRAP}\text{\_}\mathrm{CANDIDATE}\right\}.$$

Authentication failure, rejection, error, timeout, and resynchronization are responses or events rather than observable state conditions.

### 2.4. Internal Cryptographic-Context Mapping

The mapping from a message to internal cryptographic resources is represented as

(12) κ:(DataID,Src,Dst,Direction,Domain)↦(AuthServiceConfigRef,CsmJobId,KeyRef).

Here, Domain is the project-defined security domain. Configuration testing checks whether each message is mapped to its intended cryptographic context and whether messages that policy requires to remain isolated unexpectedly re-use the same resources. Context identifiers need not be transmitted on the bus; the mapping is verified from controlled configuration snapshots and internal ECU logs.

The notations used in this paper are shown in Table 2.

## 3. Threat Model and Problem Formulation

### 3.1. System Model

The test system consists of a test-control node, a sender ECU, the CAN FD bus, a receiver ECU, and an evidence store. The test-control node generates vectors, prepares receiver conditions, loads controlled configurations, injects bus traffic, and correlates logs. The sender ECU produces valid Secured I-PDUs. The receiver ECU performs freshness reconstruction, authenticator verification, state updates, and application-delivery gating. Figure 1 shows the system boundary and evidence flow.

### 3.2. Threat Model

A bus-level adversary can monitor CAN FD traffic and record valid Secured I-PDUs together with their transmission timing. The adversary can replay historical messages, modify visible on-wire fields, inject random authenticators, alter transmission periods, and increase bus load. The adversary cannot read authentication keys, modify ECU firmware or protected storage directly, bypass the SecOC module to submit messages to the application, or overwrite the receiver’s freshness state.

The laboratory environment has additional capabilities: it can load controlled configuration mutants, initialize freshness checkpoints, reset ECUs, collect authentication and freshness results, and read implementation-specific return codes. These capabilities are used only for condition setup, configuration verification, and defect injection; they are not included in the bus-level adversary model. Each test vector carries a capability label Cap(v)∈{NET,LAB}, preventing internal configuration changes from being misreported as on-wire attacks. The capability label is determined by the minimum privilege required to establish and execute a test vector, including its precondition, rather than by the nominal strength of the adversary.

The threat model assumes that SM4-CMAC meets the message-authentication objective while the key remains secret. The evaluation therefore focuses on bus injection, replay, modification of visible fields, and configuration faults, with the cryptographic primitive and key-protection mechanism treated as trusted components.

### 3.3. Real-Vehicle Scenario Adaptation and Applicability Boundary

The NET/LAB distinction reflects whether a test can be exercised through in-vehicle network access alone or requires additional engineering control. A vector is labeled NET when its required stimulus and precondition can be realized without changing protected ECU configuration or directly manipulating internal receiver state. A vector is labeled LAB when execution requires controlled configuration loading, freshness-state initialization, ECU reset, or internal implementation evidence that is normally available only in an authorized test environment.

In a production-vehicle setting, network reachability may be obtained through a diagnostic interface, a compromised in-vehicle node or gateway, or physical access to the CAN/CAN-FD network. Once such reachability is available, historical replay, re-injection of frames with modified authenticated fields, random-authenticator injection, cross-context re-use, period manipulation, and burst-load injection are represented as NET capabilities in CAST-SecOC. Here, modification of a visible on-wire field refers to capture–reconstruct–re-inject behavior or transmission of a modified frame by a controlled or compromised node. It does not assume arbitrary modification of individual bits during an ongoing CAN-FD transmission while bypassing link-layer error detection and arbitration.

The distinction is particularly relevant to freshness and rollover testing. A rollover candidate can be transmitted as an on-wire stimulus, whereas deliberately advancing or initializing the receiver near a freshness-cycle boundary to establish ROLLOVER_GUARD requires laboratory control in the present test setup. If the same receiver condition occurs naturally during vehicle operation then the corresponding oracle and evidence criteria can be applied without direct freshness state manipulation. Thus, the laboratory capability accelerates preparation of a target condition but is not interpreted as an adversary capability.

The model is applied differently across three deployment stages:

- Bench/HIL testing: both NET and LAB vectors can be executed. Laboratory controls are used to establish boundary states, inject configuration mutants, and collect internal evidence.
- Controlled real-vehicle testing: NET vectors can be exercised through an instrumented vehicle network. LAB operations may be used only as authorized pre-test preparation or instrumentation and remain explicitly labeled as laboratory capabilities.
- Production or field operation: only the subset of NET vectors compatible with operational and safety constraints is directly transferable. Vectors that require configuration replacement, freshness-state overwrite, or unrestricted internal-state access are excluded from field-level attack claims.

This separation allows the same test-vector, oracle, and evidence structure to be used from bench validation to controlled vehicle testing while keeping verification-only privileges separate from attack capabilities that may be available on an operational vehicle.

### 3.4. Test-Task Definition

A SecOC test task is defined as

(13) $$T=\langle M,S_{O},R_{FV},C,\Gamma ,U,O,E,Q\rangle ,$$

where M is the message set, $S_{O}$ the observable receiver-condition set, $R_{FV}$ the freshness-relation set, C the configuration space, Γ the perturbation-operator set, U the coverage-obligation set, O the requirement-oracle set, E the evidence set, and Q the evaluation-metric set.

A test vector is

(14) $$v=\langle m,s_{o},r_{f},c,\gamma ,\mathrm{Setup}\left(v\right),\mathrm{Stimulus}\left(v\right),\mathrm{Expected}\left(v\right),\mathrm{Cleanup}\left(v\right),\mathrm{Cap}\left(v\right)\rangle .$$

Here, Setup(v) is the condition-preparation sequence, Stimulus(v) is the test stimulus, Expected(v) specifies the behavior permitted by the requirement, and Cleanup(v) restores the test environment.

## 4. CAST-SecOC Methodology

### 4.1. Overview of CAST-SecOC

Figure 2 organizes CAST-SecOC into five functional blocks: inputs, test-vector construction, the configuration-aware SecOC testing model, execution and oracle evaluation, and outputs. Within these blocks, the workflow performs eight operational steps: requirement modeling, candidate-vector construction, feasibility filtering, coverage-based prioritization, precondition preparation, test execution, multidimensional evaluation, and evidence archiving. Test generation is constrained by the specified requirements and observable preconditions, producing on-wire inputs and controlled configuration mutations that exercise the target authentication, freshness, and configuration relations. The evaluation jointly examines the application-layer security outcome and the protocol mechanism responsible for that outcome.

The constrained construction and prioritization of test vectors are formalized in Algorithm 1, whereas the subsequent execution, multidimensional oracle evaluation, and evidence-generation procedure is summarized in Algorithm 2. Together, the two algorithms connect the configuration-aware test model to an executable workflow: Algorithm 1 produces an ordered set of feasible test vectors, and Algorithm 2 converts their observed responses into requirement-level verdicts, defect labels, and traceable evidence.
**Algorithm 1** Constrained Construction and Coverage-Gain Prioritization of Configuration-Aware Test Vectors**Require:** 
Message set M, observable-condition set $S_{O}$, freshness-relation set $R_{FV}$, configuration set C, perturbation-operator set Γ, coverage-obligation set U, and budget *B***Ensure:** 
Ordered test-vector sequence V and uncovered-obligation set $U_{\mathrm{uncov}}$  1:$V_{\mathrm{cand}}\leftarrow \emptyset$                                         ▹ Initialize candidate vectors.  2:**for all** $\left(m,s_{o},r_{f},c,\gamma \right)\in M\times S_{O}\times R_{FV}\times C\times \Gamma$ **do**                       ▹ Enumerate test combinations.  3:    **if** $\mathrm{Compatible}(m,s_{o},r_{f},c,\gamma )$ **then**                                ▹ Keep feasible combinations.  4:        $v\leftarrow \mathrm{Instantiate}(m,s_{o},r_{f},c,\gamma )$                                 ▹ Construct a test vector.  5:        Expected(v)←RequirementOracle(v)                          ▹ Assign expected behavior.  6:        Cov(v)←CoverageObligations(v)                           ▹ Determine covered obligations.  7:        $V_{\mathrm{cand}}\leftarrow V_{\mathrm{cand}}\cup \left\{v\right\}$                                       ▹ Add the feasible vector.  8:    **end if**  9:**end for**10:Reduce equivalent candidate templates using $K_{\mathrm{eq}}$                        ▹ Remove semantic redundancy.11:V←〈〉, $U_{\mathrm{uncov}}\leftarrow U$                                   ▹ Initialize selection and coverage.12:**while** |V|<B, $V_{\mathrm{cand}}\neq \emptyset$, and $U_{\mathrm{uncov}}\neq \emptyset$ **do**                       ▹ Continue while selection is possible.13:                                              ▹ Select the highest-gain vector.14:    Select $v^{★}$ with maximum $\mathrm{Score}(v|U_{\mathrm{uncov}})$; break ties by ascending VectorID15:    Append $v^{★}$ to V                                         ▹ Add to execution order.16:    $U_{\mathrm{uncov}}\leftarrow U_{\mathrm{uncov}}\setminus \mathrm{Cov}\left(v^{★}\right)$                               ▹ Update uncovered obligations.17:    $V_{\mathrm{cand}}\leftarrow V_{\mathrm{cand}}\setminus \left\{v^{★}\right\}$                                    ▹ Remove the selected vector.18:**end while**19:**return** $\left(V,U_{\mathrm{uncov}}\right)$                                   ▹ Return vectors and residual coverage.

**Algorithm 2** CAST-SecOC Execution, Multidimensional Verdict Assignment, and Evidence Generation
**Require:** 
Ordered test-vector sequence V, configuration set C, and requirement oracle *O***Ensure:** 
Metric set Q, defect set D, and evidence set E                                        ▹ Synchronize execution and evidence.  1:Initialize CANoe, Communication Access Programming Language (CAPL), the ECU log interface, and a common time base  2:D←∅, E←∅                                            ▹ Initialize result sets.  3:**for** i=1 to |V| **do**                                          ▹ Follow the prioritized order.  4:    **if** i>1 **then**  5:        Execute $\mathrm{Cleanup}(v_{i-1})$                                   ▹ Remove residual test effects.  6:    **end if**  7:    Restore the baseline checkpoint                                 ▹ Restore a controlled state.  8:    Load configuration $c_{i}$; record its hash and loading result                        ▹ Bind execution to $c_{i}$.                                         ▹ Prepare and confirm the precondition.  9:    Execute $\mathrm{Setup}(v_{i})$ and verify the evidence for the target condition10:    **if** the target condition cannot be confirmed **then**                      ▹ Handle unverified preconditions.                                            ▹ Mark evidence as incomplete.11:        Set $\eta_{i}\leftarrow \mathrm{INCOMPLETE}$ and $verdict_{i}\leftarrow \mathrm{INCONCLUSIVE}$                                               ▹ Preserve available evidence.12:        Store the vector, configuration, setup trace, incomplete response, and available logs in E13:        **continue**                                         ▹ Skip stimulus execution.14:    **end if**                                         ▹ Execute stimulus and collect evidence.15:    Inject $\mathrm{Stimulus}(v_{i})$; collect the bus trace and internal ECU logs16:    Construct response $r_{i}$ and compute $verdict_{i}\leftarrow O\left(v_{i},r_{i}\right)$                     ▹ Apply the requirement oracle.17:    **if** $verdict_{i}=\mathrm{FAIL}$ **then**                                  ▹ Localize conclusive failures.18:        Generate defect labels using Equations (38)–(48)                          ▹ Identify the defect type.19:        $D\leftarrow D\cup \{\mathrm{Defect}\left(v_{i},c_{i},r_{i}\right)\}$                               ▹ Record the detected defect.20:    **end if**21:    Store $\varepsilon (v_{i})$ in E                                       ▹ Archive execution evidence.22:
**end for**
                                              ▹ Compute evaluation metrics.23:Compute IAR, FRR, DMR, IR, NPV, $MS_{\mathrm{cfg}}$, dimension-specific mutation scores, dimensional coverage, and latency distributions24:**return**(Q,D,E)                                 ▹ Return metrics, defects, and evidence.


### 4.2. Message and Configuration Models

A message $m_{i}$ is represented as

(15) $$m_{i}=\langle {\mathrm{CANID}}_{i},{\mathrm{DataID}}_{i},{\mathrm{Src}}_{i},{\mathrm{Dst}}_{i},L_{i},T_{i},D_{i},R_{i},{\mathrm{Sem}}_{i},{\mathrm{Critical}}_{i}\rangle ,$$

where $L_{i}$ is the Authentic I-PDU length, $T_{i}$ the transmission period, $D_{i}$ the end-to-end deadline, $R_{i}$ the risk level, ${\mathrm{Sem}}_{i}$ the message semantics, and ${\mathrm{Critical}}_{i}$ the set of safety-critical byte or signal positions.

A configuration $c_{r}$ is represented as

(16) $$c_{r}=\langle \ell_{r},\lambda_{r},b_{r},W_{r},\rho_{r},A_{r},\kappa_{r},\pi_{r}^{\mathrm{timeout}},\pi_{r}^{\mathrm{persist}}\rangle ,$$

where $\rho_{r}$ denotes the rollover and resynchronization policy, $\pi_{r}^{\mathrm{timeout}}$ the verification-timeout policy, and $\pi_{r}^{\mathrm{persist}}$ the freshness-state persistence policy. In addition to authenticator length and acceptance-window size, Equation (16) explicitly includes the authenticated region, cryptographic context, timeout handling, and state persistence, so that each configuration mutant can be linked to a specific security requirement. The nine configuration dimensions in Equation (16) are also used as the basis for systematic configuration mutation. Let

$$D_{C}=\left\{\ell ,\lambda ,b,W,\rho ,A,\kappa ,\pi^{\mathrm{timeout}},\pi^{\mathrm{persist}}\right\}.$$

For a baseline configuration $c_{0}$, a first-order configuration mutant changes exactly one dimension $d\in D_{C}$ while keeping all remaining dimensions unchanged. Supported non-baseline values or policies of that dimension are enumerated before test execution. Mutants that cannot be loaded by the evaluated platform are recorded as load-rejected, while mutants with no observable semantic difference from another generated configuration are classified as equivalent and removed from the mutation-score denominator.

Importantly, mutant generation is performed independently of the defect criteria defined in Section 4.5.9. The mutation set is therefore derived from the configuration space itself rather than from a predefined set of faults that the oracle is explicitly designed to detect.

The perturbation-operator set is

(17) $$\begin{array}{cc} \Gamma =\;\{ & \mathrm{NONE},\mathrm{TAMPER}\text{\_}\mathrm{AUTH},\mathrm{FORGE}\text{\_}\mathrm{MAC},\\ \; & \mathrm{REPLAY},\mathrm{CROSS}\text{\_}\mathrm{CONTEXT},\mathrm{BURST}\text{\_}\mathrm{INJECTION}\}. \end{array}$$

Window-boundary and rollover cases are represented through the freshness-relation dimension $R_{FV}$, whereas authenticator length, authenticated-region selection, and context mapping are represented in the configuration space C.

High-dimensional chaotic and memristive-neuron models can also provide pseudo-random sequences for increasing perturbation diversity [30,31]. Within CAST-SecOC, such sequences could be used to parameterize perturbation positions, forged field or authenticator values, replay intervals, operator ordering, or burst-injection spacing. These parameters would still be subject to the compatibility constraints, coverage obligations, and requirement-level oracle of CAST-SecOC. The current experimental campaign does not employ chaotic sequence generators; they are considered a possible extension for future fuzzing and robustness testing. The coverage-obligation set U comprises observable-condition obligations $U_{S}$, freshness-relation obligations $U_{F}$, configuration-boundary obligations $U_{C}$, perturbation obligations $U_{\Gamma }$, requirement obligations $U_{R}$, and message-risk obligations $U_{M}$.

### 4.3. Observable-Condition Preparation and Reachability Constraints

A precondition is included in a test vector only when it can be established through an executable preparation procedure and independently confirmed by the available evidence. CAST-SecOC uses two observable receiver conditions corresponding to the normal verification path and the rollover-gating path, respectively.

Under the SYNCED condition, valid messages are transmitted using the baseline configuration until the receiver’s last-accepted freshness value advances steadily. Successful preparation is confirmed by authentication success, freshness verification success, application-layer delivery, and monotonic advancement of the last-accepted value. After the corresponding test is completed, the receiver is restored to the baseline checkpoint.

The ROLLOVER_GUARD condition is prepared by advancing the full freshness value to the vicinity of $2^{b}-1$ and then submitting a low-value rollover candidate. The condition is considered successfully established when the receiver recognizes the rollover candidate, prevents application-layer delivery, and records an authenticated resynchronization or cycle-confirmation action. After execution, the baseline freshness cycle is restored to prevent the rollover state from affecting subsequent test vectors.

Historical and out-of-window inputs are represented by the freshness relation $r_{f}$; resynchronization behavior is recorded by $a_{\mathrm{resync}}$; and receiver state changes are recorded by $u_{\mathrm{state}}$. Loss of synchronization is not treated as an independent observable receiver condition unless it can be established and confirmed through the available evidence chain. A candidate combination is instantiated only when

(18) $$\mathrm{Compatible}(m,s_{o},r_{f},c,\gamma )=\mathrm{TRUE}.$$

The feasibility filter checks message length and field positions, the relation between the perturbation target and authenticated region, the executable setup sequence, the freshness relation, configuration-loading capability, and the observation points required by the oracle. A combination enters the execution set only after its setup and observability constraints have been satisfied.

### 4.4. Coverage-Gain-Driven Test-Vector Generation

Let Cov(v) be the set of obligations covered by candidate vector *v*. Given the currently uncovered set $U_{\mathrm{uncov}}$, the selection score is

(19) $$\mathrm{Score}\left(v|U_{\mathrm{uncov}}\right)=w_{g}\frac{|\mathrm{Cov}\left(v\right)\cap U_{\mathrm{uncov}}|}{|U_{\mathrm{uncov}}|}+w_{b}I_{\mathrm{boundary}}\left(v\right)+w_{r}\mathrm{Risk}\left(m_{v}\right)-w_{t}\mathrm{Cost}\left(v\right),$$

where $w_{g}+w_{b}+w_{r}+w_{t}=1$, and $m_{v}$ denotes the message associated with vector *v*. The cost term combines condition-preparation effort, configuration-switching effort, and execution time. Under fixed weights and a predefined tie-breaking rule, the score prioritizes vectors that increase coverage, exercise configuration boundaries, target higher-risk messages, and reduce execution cost. Ties are resolved by ascending VectorID, which makes the execution order reproducible.

Equivalent-vector reduction is applied to semantic test templates rather than to their observed response tuples. For a candidate vector *v*, we define its equivalence key as

(20) $$K_{\mathrm{eq}}\left(v\right)=\langle m,\;s_{o},\;r_{f},\;c,\;\gamma ,\;t\left(v\right),\;\chi \left(v\right),\;\mathrm{Cov}\left(v\right)\rangle ,$$

where t(v) denotes the semantic perturbation target, such as an authenticated payload byte or signal position, the authenticator field, a freshness boundary, or a source–destination/context pair. Two candidate templates $v_{a}$ and $v_{b}$ are equivalent, written $v_{a}∼v_{b}$, only when $K_{\mathrm{eq}}\left(v_{a}\right)=K_{\mathrm{eq}}\left(v_{b}\right)$ and their remaining differences are concrete values or execution metadata that do not change the modeled receiver semantics.

Consequently, vectors that exercise different messages, perturbation targets, freshness relations, configuration values, context mappings, requirement classes, or coverage obligations are not merged merely because they later produce the same authentication, freshness, delivery, and state-update outcome.

The reduction retains one template representative from each equivalence class for coverage-based selection. A retained template may subsequently be instantiated multiple times using different concrete payload values, freshness checkpoints, or repeated executions. Let G denote the retained equivalence-class representatives and $n_{g}$ the number of concrete executions instantiated from class *g*. The campaign size is therefore

(21) $$N_{\mathrm{exec}}=\sum_{g\in G}n_{g},\;N_{\mathrm{class}}=\left|G\right|.$$

Thus, $N_{\mathrm{exec}}$ quantifies concrete executions, whereas $N_{\mathrm{class}}$ quantifies distinct input-semantic test classes.

Algorithm 1 has two stages. Candidate construction enumerates combinations of messages, observable receiver conditions, freshness relations, SecOC configurations, and perturbation operators. The compatibility predicate removes combinations that cannot be instantiated under the protocol semantics, available configuration controls, or observation points. Each feasible combination is converted into a test vector, assigned an expected response by the requirement oracle in Section 4.5, and associated with its coverage obligations. Equivalent candidate templates are grouped using Equation (20), and one representative from each class enters coverage-based prioritization. This reduction removes semantic duplication at the template level; it does not collapse repeated concrete executions instantiated from a retained class. Consequently, the number of executed vectors and the number of retained equivalence classes are reported separately in the experimental results.

The prioritization stage repeatedly selects the highest-scoring remaining vector. After each selection, the covered obligations are removed from $U_{\mathrm{uncov}}$, and the remaining scores are evaluated against the updated set. The procedure terminates when the budget is exhausted, all obligations are covered, or no candidate remains. Its outputs are an ordered executable sequence and the residual obligations that could not be covered under the available budget and configuration constraints.

### 4.5. Response Representation, Requirement Oracle, and Defect Criteria

The CAST-SecOC oracle does not reduce a response to a single Accept, Reject, or Timeout label. Instead, it evaluates a multidimensional response that captures application delivery, internal verification decisions, state changes, execution status, and latency. This representation distinguishes correct blocking of an invalid message from an unintended rejection path, authenticated recovery, and incomplete evidence.

#### 4.5.1. Multidimensional Response and Evidence Completeness

The receiver response to test vector *v* is

(22) $$r\left(v\right)=\langle z_{\mathrm{auth}},z_{\mathrm{fresh}},d_{\mathrm{app}},a_{\mathrm{resync}},u_{\mathrm{state}},z_{\mathrm{exec}},e,\tau ,\eta \rangle ,$$

where:

- $z_{\mathrm{auth}}\in \left\{\mathrm{PASS},\mathrm{FAIL},\mathrm{NA}\right\}$ is the authenticator-verification result;
- $z_{\mathrm{fresh}}\in \left\{\mathrm{PASS},\mathrm{FAIL},\mathrm{NA}\right\}$ is the freshness-verification result;
- $d_{\mathrm{app}}\in \left\{0,1\right\}$ indicates whether the Authentic I-PDU was delivered to the application;
- $a_{\mathrm{resync}}\in \left\{\mathrm{NONE},\mathrm{REQUESTED},\mathrm{AUTHENTICATED},\mathrm{UNAUTHENTICATED}\right\}$ records the resynchronization action;
- $u_{\mathrm{state}}\in \left\{\mathrm{UNCHANGED},\mathrm{UPDATED}\text{\_}\mathrm{AUTH},\mathrm{UPDATED}\text{\_}\mathrm{UNAUTH},\mathrm{UNKNOWN}\right\}$ records whether freshness state was updated and whether the update had an authenticated basis;
- $z_{\mathrm{exec}}\in \left\{\mathrm{NORMAL},\mathrm{ERROR},\mathrm{TIMEOUT}\right\}$ records the execution status;
- *e* stores the corresponding implementation-specific error or timeout code, when present;
- τ is the measured end-to-end latency from the sender-side message-release timestamp to final application delivery or rejection. The sender-side message-release timestamp is recorded immediately before SecOC authentication generation, so the measured end-to-end latency includes sender-side cryptographic processing.
- η∈{COMPLETE,INCOMPLETE} indicates whether the evidence required by the oracle is complete.

For invalid messages, $d_{\mathrm{app}}$ is the primary security observation. An authentication or freshness failure satisfies the intended security property only if the anomalous input does not reach the application. An error or timeout is not automatically counted as a security pass: an error must reflect controlled handling, and a timeout must be assessed against the message deadline and the available evidence.

#### 4.5.2. Vector Classes and Verdict Structure

The message-level requirement class is

(23) $$\chi_{\mathrm{msg}}\left(v\right)\in \left\{\mathrm{VALID},\mathrm{TAMPER},\mathrm{REPLAY},\mathrm{CONTEXT},\mathrm{BOUNDARY}\text{\_}\mathrm{OUT},\mathrm{ROLLOVER}\right\}.$$

Configuration tests are classified separately as

(24) $$\begin{array}{cc} \chi_{\mathrm{cfg}}\left(v\right) & \in \{\mathrm{SYSTEMATIC}\text{\_}\mathrm{CONFIG}\text{\_}\mathrm{MUTATION},\mathrm{PARAMETER}\text{\_}\mathrm{POLICY}\},\\ L(v) & \in \{\mathrm{NORMAL},\mathrm{HIGH}\}. \end{array}$$

where L(v) is an execution-condition label rather than a message-level oracle class.

For a message-level vector, the oracle combines a security-outcome predicate $\psi_{\chi_{\mathrm{msg}}\left(v\right)}$ with a mechanism-path predicate $\omega_{\chi_{\mathrm{msg}}\left(v\right)}$:

(25) $$O\left(v,r\right)=\left\{\begin{array}{cc} \mathrm{INCONCLUSIVE}, & \eta =\mathrm{INCOMPLETE},\\ \mathrm{PASS}, & \eta =\mathrm{COMPLETE}\wedge \psi_{\chi_{\mathrm{msg}}\left(v\right)}\left(v,r\right)\wedge \omega_{\chi_{\mathrm{msg}}\left(v\right)}\left(v,r\right),\\ \mathrm{FAIL}, & \mathrm{otherwise}. \end{array}\right.$$

The outcome predicate determines whether an invalid input crossed the application boundary or a valid input was rejected. The mechanism predicate checks whether authentication, freshness handling, recovery, and state updates followed the path required for the vector. Timing for a valid message is recorded independently as

(26) $$T\left(v,r\right)=\left\{\begin{array}{cc} \mathrm{ON}\text{\_}\mathrm{TIME}, & d_{\mathrm{app}}=1\wedge \tau \leq D\left(v\right),\\ \mathrm{LATE}, & d_{\mathrm{app}}=1\wedge \tau >D\left(v\right),\\ \mathrm{NOT}\text{\_}\mathrm{DELIVERED}, & d_{\mathrm{app}}=0. \end{array}\right.$$

A late delivery is therefore not conflated with a false rejection.

#### 4.5.3. Valid-Message Oracle

For a valid vector, the outcome and mechanism predicates are

(27) $$\psi_{\mathrm{VALID}}\left(v,r\right)=\left[d_{\mathrm{app}}=1\right]\wedge \left[z_{\mathrm{exec}}=\mathrm{NORMAL}\right],$$

(28) $$\omega_{\mathrm{VALID}}\left(v,r\right)=\left[z_{\mathrm{auth}}=\mathrm{PASS}\right]\wedge \left[z_{\mathrm{fresh}}=\mathrm{PASS}\right]\wedge \left[u_{\mathrm{state}}\in \left\{\mathrm{UNCHANGED},\mathrm{UPDATED}\text{\_}\mathrm{AUTH}\right\}\right].$$

Failure to deliver a valid message constitutes a functional failure of the SecOC protection chain. A valid message delivered after its deadline is classified as LATE and counted separately by the availability metrics.

#### 4.5.4. Tampering and Authenticator-Forgery Oracle

For a vector that modifies only an authenticated payload region or the authenticator field, the anomalous message must not reach the application or induce an unauthenticated state update. The expected mechanism is authentication failure while freshness remains consistent with the baseline sequence:

(29) $$\psi_{\mathrm{TAMPER}}\left(v,r\right)=\left[d_{\mathrm{app}}=0\right]\wedge \left[u_{\mathrm{state}}\neq \mathrm{UPDATED}\text{\_}\mathrm{UNAUTH}\right],$$

(30) $$\omega_{\mathrm{TAMPER}}\left(v,r\right)=\left[z_{\mathrm{auth}}=\mathrm{FAIL}\right]\wedge \left[z_{\mathrm{fresh}}=\mathrm{PASS}\right].$$

This mechanism predicate confirms that authenticator verification, rather than an unrelated earlier condition, blocked the vector.

#### 4.5.5. Replay and Historical-Freshness Oracle

A historical Secured I-PDU may carry a valid authenticator. The corresponding predicates are

(31) $$\psi_{\mathrm{REPLAY}}\left(v,r\right)=\left[d_{\mathrm{app}}=0\right]\wedge \left[u_{\mathrm{state}}\neq \mathrm{UPDATED}\text{\_}\mathrm{UNAUTH}\right],$$

(32) $$\omega_{\mathrm{REPLAY}}\left(v,r\right)=\left[z_{\mathrm{auth}}=\mathrm{PASS}\right]\wedge \left[z_{\mathrm{fresh}}=\mathrm{FAIL}\right].$$

This oracle distinguishes a valid authenticator with invalid freshness from an ordinary authentication failure.

#### 4.5.6. Cross-Context Oracle

Inputs crossing a message, direction, or security-domain boundary must be blocked by cryptographic-context isolation:

(33) $$\psi_{\mathrm{CONTEXT}}\left(v,r\right)=\left[d_{\mathrm{app}}=0\right]\wedge \left[u_{\mathrm{state}}\neq \mathrm{UPDATED}\text{\_}\mathrm{UNAUTH}\right],$$

(34) $$\omega_{\mathrm{CONTEXT}}\left(v,r\right)=\left[z_{\mathrm{auth}}=\mathrm{FAIL}\right]\wedge \left[z_{\mathrm{fresh}}=\mathrm{PASS}\right].$$

#### 4.5.7. Window-Boundary and Rollover Oracles

For an out-of-window candidate, the message must not be delivered and must not trigger an unauthenticated state update. The expected mechanism is freshness rejection or an explicit policy rejection. Let $K_{\mathrm{policy}\text{\_}\mathrm{reject}}$ denote the implementation-specific codes assigned to such policy decisions:

(35) $$\begin{array}{cc} \psi_{\mathrm{BOUNDARY}\text{\_}\mathrm{OUT}}\left(v,r\right) & =\left[d_{\mathrm{app}}=0\right]\wedge \left[u_{\mathrm{state}}\neq \mathrm{UPDATED}\text{\_}\mathrm{UNAUTH}\right],\\ \omega_{\mathrm{BOUNDARY}\text{\_}\mathrm{OUT}}\left(v,r\right) & =\left[z_{\mathrm{fresh}}=\mathrm{FAIL}\right]\vee \left(\left[z_{\mathrm{exec}}=\mathrm{ERROR}\right]\wedge \left[e\in K_{\mathrm{policy}\text{\_}\mathrm{reject}}\right]\right). \end{array}$$

Authentication may also fail in implementations that evaluate or report the two checks jointly; the freshness or policy-rejection condition remains the required evidence for this class.

For a rollover candidate, local authentication and freshness checks may both pass, but the candidate must remain gated until the receiver obtains authenticated evidence for the new cycle:

(36) $$\begin{array}{cc} \psi_{\mathrm{ROLLOVER}}\left(v,r\right) & =\left[d_{\mathrm{app}}=0\right]\wedge \left[u_{\mathrm{state}}\neq \mathrm{UPDATED}\text{\_}\mathrm{UNAUTH}\right],\\ \omega_{\mathrm{ROLLOVER}}\left(v,r\right) & =\left[z_{\mathrm{auth}}=\mathrm{PASS}\right]\wedge \left[z_{\mathrm{fresh}}=\mathrm{PASS}\right]\wedge \left[a_{\mathrm{resync}}=\mathrm{AUTHENTICATED}\right]. \end{array}$$

#### 4.5.8. Systematic Configuration Mutation and High-Load Execution

Let $C_{\mu }^{\mathrm{sys}}$ denote the systematic first-order mutant set generated from the nine dimensions in Equation (16), and let $V_{\mathrm{reg}}$ denote the fixed regression-vector set constructed before the mutation campaign. No additional vector is introduced for a particular mutant after its configuration change is known.

For each loadable, non-equivalent mutant $c_{\mu }$, the applicable regression vectors are re-executed under the mutated configuration. A mutant is killed when at least one vector that passes under the baseline configuration produces a requirement-level failure under the mutant:

$$\mathrm{Killed}\left(c_{\mu }\right)=1⟺\exists v\in V_{\mathrm{reg}}:O_{c_{0}}\left(v,r_{c_{0}}\left(v\right)\right)=\mathrm{PASS}\wedge O_{c_{\mu }}\left(v,r_{c_{\mu }}\left(v\right)\right)=\mathrm{FAIL}.$$

An executable non-equivalent mutant survives when no vector in the fixed regression suite distinguishes it from the baseline:

$$\mathrm{Survived}\left(c_{\mu }\right)=1⟺\mathrm{Killed}\left(c_{\mu }\right)=0.$$

Load-rejected and equivalent mutants are reported separately and are excluded from the mutation-score denominator. A surviving mutant is retained as an explicit adequacy finding because it identifies configuration behavior that the current regression suite does not distinguish.

A vector executed under high load retains its underlying message-level class:

(37) $$O_{\mathrm{HIGH}}\left(v,r\right)=O_{\chi_{\mathrm{msg}}\left(v\right)}\left(v,r\right).$$

The security semantics therefore remain unchanged, while timing for valid vectors is still governed by Equation (26).

#### 4.5.9. Configuration-Defect Localization Criteria

When O(v,r)=FAIL and the evidence is complete, CAST-SecOC applies the following executable defect criteria:

(1) Omission from the authenticated region.

Let $j\in {\mathrm{Critical}}_{i}$ be a safety-critical byte position in message $m_{i}$. If *j* lies outside the authenticated byte-index set and a modification at that position still passes authentication and reaches the application, an authenticated-region omission is reported:

(38) $$D_{\mathrm{area}}=1⟺j\in {\mathrm{Critical}}_{i}\setminus I_{i}\wedge z_{\mathrm{auth}}=\mathrm{PASS}\wedge d_{\mathrm{app}}=1.$$

(2) Fail-open delivery after authentication failure:

(39) $$D_{\mathrm{failopen}}=1⟺z_{\mathrm{auth}}=\mathrm{FAIL}\wedge d_{\mathrm{app}}=1.$$

(3) Acceptance of a historical freshness value.

Within one freshness cycle, delivery of a reconstructed value no greater than the last accepted value constitutes rollback acceptance:

(40) $$D_{\mathrm{rollback}}=1⟺\hat{FV}\leq FV_{\mathrm{last}}\wedge d_{\mathrm{app}}=1.$$

(4) Acceptance of an out-of-window candidate.

Outside a rollover condition, delivery with $\hat{FV}-FV_{\mathrm{last}}>W$ indicates failure of the window constraint:

(41) $$D_{\mathrm{window}}=1⟺s_{o}\neq \mathrm{ROLLOVER}\text{\_}\mathrm{GUARD}\wedge \left(\hat{FV}-FV_{\mathrm{last}}>W\right)\wedge d_{\mathrm{app}}=1.$$

(5) Missing rollover gate:

(42) $$D_{\mathrm{rollover}}=1⟺s_{o}=\mathrm{ROLLOVER}\text{\_}\mathrm{GUARD}\wedge d_{\mathrm{app}}=1\wedge a_{\mathrm{resync}}\neq \mathrm{AUTHENTICATED}.$$

(6) Unauthenticated state update:

(43) $$D_{\mathrm{unauth}\text{\_}\mathrm{update}}=1⟺u_{\mathrm{state}}=\mathrm{UPDATED}\text{\_}\mathrm{UNAUTH}.$$

(7) Insufficient cryptographic-context isolation.

If policy requires messages $m_{i}$ and $m_{j}$ to use separate cryptographic contexts, but both map to the same context and a cross-context witness is delivered, the mapping is insufficiently isolated:

(44) $$\begin{array}{cc} D_{\mathrm{context}}=1⟺\; & \mathrm{PolicySeparate}\left(m_{i},m_{j}\right)=1\wedge \kappa \left(m_{i}\right)=\kappa \left(m_{j}\right)\\ \; & \wedge d_{\mathrm{app}}\left(v_{i\to j}\right)=1. \end{array}$$

(8) Non-compliant authenticator-length policy.

Let *q* be the upper bound on online forgery attempts and θ the maximum permitted probability of at least one successful random forgery. For an *ℓ*-bit authenticator,

(45) $$P_{\mathrm{forge}}\left(q,\ell \right)=1-{\left(1-2^{-\ell }\right)}^{q}.$$

Let $a_{\mathrm{cfg}}\left(c\right)\in \left\{0,1\right\}$ indicate whether configuration *c* is accepted for loading or execution. An accepted policy violation is reported when

(46) $$D_{\mathrm{authlen}}\left(c\right)=1⟺P_{\mathrm{forge}}\left(q,\ell \left(c\right)\right)>\theta \wedge a_{\mathrm{cfg}}\left(c\right)=1.$$

The required authenticator length is therefore determined jointly by the attack-attempt budget and the tolerated risk threshold.

(9) False rejection of a valid message:

(47) $$D_{\mathrm{false}\text{\_}\mathrm{reject}}=1⟺v\in V_{\mathrm{valid}}\wedge d_{\mathrm{app}}=0.$$

(10) Deadline miss:

(48) $$D_{\mathrm{deadline}}=1⟺v\in V_{\mathrm{valid}}\wedge d_{\mathrm{app}}=1\wedge \tau >D\left(v\right).$$

Table 3 summarizes the principal requirements for the six message-level classes.

High load is handled as an execution condition rather than an additional oracle class. The underlying message-level row remains applicable, and deadline misses for valid traffic are recorded independently.

#### 4.5.10. Binary Baseline Oracle for Ablation

To isolate the diagnostic contribution of the multidimensional oracle, we define an application-only binary baseline using the same execution records. For a response *r*, the binary observation is

(49) $$B\left(r\right)=\left\{\begin{array}{cc} \mathrm{INCONCLUSIVE}, & \eta =\mathrm{INCOMPLETE},\\ \mathrm{ACCEPT}, & \eta =\mathrm{COMPLETE}\wedge d_{\mathrm{app}}=1,\\ \mathrm{REJECT}, & \eta =\mathrm{COMPLETE}\wedge d_{\mathrm{app}}=0. \end{array}\right.$$

The binary oracle does not inspect $z_{\mathrm{auth}}$, $z_{\mathrm{fresh}}$, $a_{\mathrm{resync}}$, $u_{\mathrm{state}}$, $z_{\mathrm{exec}}$, *e* or the timing verdict. It therefore represents the minimum application-boundary observation: whether the Authentic I-PDU was delivered.

The ablation is applied to message-level execution records only. Authenticator-length policy cases are excluded because their verdict is defined over configuration-policy compliance rather than application delivery. No ECU execution is repeated for this comparison; the existing evidence records are re-scored offline using Equation (49).

### 4.6. Evaluation Metrics

The metrics separately characterize message-level security, valid-message availability, evidence completeness, configuration-policy enforcement, systematic configuration-mutation adequacy, and test coverage. Rejection, latency, execution errors, and configuration findings are not collapsed into a generic detection rate.

The invalid-acceptance rate is

(50) $$\mathrm{IAR}=\frac{N_{\mathrm{invalid},\mathrm{delivered}}}{N_{\mathrm{invalid},\mathrm{conclusive}}}.$$

The false-rejection rate counts valid inputs that are not delivered:

(51) $$\mathrm{FRR}=\frac{N_{\mathrm{valid},\mathrm{not}\;\mathrm{delivered}}}{N_{\mathrm{valid},\mathrm{conclusive}}}.$$

For delivered valid messages, the deadline-miss rate is

(52) $$\mathrm{DMR}=\frac{N_{\mathrm{valid},\mathrm{delivered}\;\mathrm{after}\;\mathrm{deadline}}}{N_{\mathrm{valid},\mathrm{delivered}}}.$$

The inconclusive rate is

(53) $$\mathrm{IR}=\frac{N_{\mathrm{inconclusive}}}{N_{\mathrm{executed}}}.$$

Parameter policies, such as authenticator-length constraints, are not online message attacks. CAST-SecOC therefore reports the number of execution records in which a policy-violating configuration is accepted for loading or execution:

(54) $$NPV=N_{\mathrm{policy}\text{-}\mathrm{violation},\mathrm{accepted}\;\mathrm{records}}.$$

The corresponding ConfigID and policy basis are retained; no rate is computed over a mixture of compliant and non-compliant configurations.

For the executable, non-equivalent systematic configuration mutants, the configuration mutation score is defined as

(55) $$MS_{\mathrm{cfg}}=\frac{N_{\mathrm{killed}}}{N_{\mathrm{killed}}+N_{\mathrm{survived}}}.$$

To identify differences in fault-detection sensitivity among the nine configuration dimensions, a dimension-specific mutation score is defined as

(56) $$MS_{d}=\frac{N_{\mathrm{killed},d}}{N_{\mathrm{killed},d}+N_{\mathrm{survived},d}},\;d\in D_{C}.$$

Load-rejected and equivalent mutants are reported separately and are excluded from the denominator of $MS_{\mathrm{cfg}}$. Therefore, configuration-loader rejection and semantic equivalence are not counted as evidence of fault-detection capability.

Observable receiver conditions are reported as the executed set $S_{O,\mathrm{cov}}$, rather than divided by an artificially defined state universe. For dimensions with a predefined finite universe, coverage is

(57) $$\begin{array}{cc} C_{\mathrm{fresh}}\; & =\frac{|R_{FV,\mathrm{cov}}|}{|R_{FV}|},\;C_{\mathrm{config}}=\frac{|C_{\mathrm{boundary},\mathrm{cov}}|}{|C_{\mathrm{boundary}}|},\\ C_{\mathrm{operator}}\; & =\frac{|\Gamma_{\mathrm{cov}}|}{\left|\Gamma \right|},\;C_{\mathrm{requirement}}=\frac{|U_{R,\mathrm{cov}}|}{|U_{R}|}. \end{array}$$

Each dimension is reported separately so that mutation-based configuration adequacy and coverage of observable conditions, freshness relations, configuration boundaries, perturbation operators, and requirement classes remain explicit. Latency is summarized by the mean, standard deviation, median, P95, and P99. For an observed event rate, the two-sided 95% Wilson confidence interval is calculated as

(58) $${\mathrm{CI}}_{0.95}=\frac{\hat{p}+\frac{z^{2}}{2n}\pm z\sqrt{\frac{\hat{p}\left(1-\hat{p}\right)}{n}+\frac{z^{2}}{4n^{2}}}}{1+\frac{z^{2}}{n}},\;z=z_{0.975}=1.96,$$

where $\hat{p}=k/n$, *k* is the number of observed events, and *n* is the number of observations. Equation (58) is used when both the lower and upper confidence limits are reported, such as for the observed high-load deadline-miss rate.

We therefore use the one-sided 95% Wilson upper confidence bound

(59) $$U_{0.95}=\frac{\hat{p}+\frac{z^{2}}{2n}+z\sqrt{\frac{\hat{p}\left(1-\hat{p}\right)}{n}+\frac{z^{2}}{4n^{2}}}}{1+\frac{z^{2}}{n}},\;z=z_{0.95}=1.645.$$

When no event is observed, i.e., k=0 and $\hat{p}=0$, Equation (59) reduces to

(60) $$U_{0.95}=\frac{z^{2}}{n+z^{2}},\;z=1.645.$$

### 4.7. Test Execution and Evidence Generation

Each test vector is executed from a controlled baseline to limit unintended state coupling between consecutive tests. Before stimulus injection, the corresponding configuration is loaded and the required observable receiver condition is prepared and independently confirmed. CAPL performs message injection and timing control, the ECU reports authentication, freshness, state-update, execution-status, and error information, and the Python 3.10 controller correlates the bus trace with internal logs.

An evidence record associated with vector *v* is

(61) $$\begin{array}{cc} \varepsilon (v)=\;\langle & \mathrm{VectorID},\mathrm{ConfigID},\mathrm{ConfigHash},\mathrm{StateConditionBefore},\mathrm{SetupTrace},\\ \; & \mathrm{Stimulus},z_{\mathrm{auth}},z_{\mathrm{fresh}},d_{\mathrm{app}},a_{\mathrm{resync}},u_{\mathrm{state}},z_{\mathrm{exec}},e,\tau ,\\ \; & \mathrm{TimingVerdict},\mathrm{Verdict},\mathrm{DefectLabel},\mathrm{BusTrace},\mathrm{EcuLog},\mathrm{Timestamp}\rangle , \end{array}$$

and the complete evidence set is

(62) E={ε(v)∣v∈V}.

This structure links the requirement, test input, precondition, configuration version, ECU response, timing result, and defect conclusion.

Algorithm 2 summarizes test execution, oracle evaluation, defect classification, and evidence generation.

Algorithm 2 processes the vectors in their prioritized order. The baseline checkpoint is restored before each execution, and cleanup is applied only when a preceding vector exists. If the target precondition cannot be confirmed, the vector receives an INCONCLUSIVE verdict and the available setup evidence is stored before execution continues. For a conclusively prepared vector, the controller injects the stimulus, correlates the CAN FD trace with ECU logs, constructs the multidimensional response, and applies the requirement-level oracle. Only a FAIL verdict triggers defect classification. PASS, FAIL, and INCONCLUSIVE executions all remain traceable through the evidence set.

## 5. Experimental Design and Results

### 5.1. Experimental Design

The evaluation addressed four aspects of CAST-SecOC: message-level security behavior, configuration-fault localization, mutation-based test adequacy, and execution overhead. The security experiments covered valid communication, payload tampering within the authenticated region, authenticator forgery, historical replay, freshness-window boundaries, counter rollover, cryptographic-context mapping, and high-load injection.

Configuration-test adequacy was evaluated using a systematic first-order mutation campaign derived from all nine configuration dimensions in Equation (16). One configuration dimension was changed at a time while the remaining dimensions were kept at their baseline settings. In total, 24 mutants were generated. Two were rejected during configuration loading and two were classified as equivalent, leaving 20 executable non-equivalent mutants for mutation-score evaluation. The same fixed regression-vector set was applied across these mutants, without introducing mutant-specific witness vectors.

Authenticator-length policy testing remained a separate parameter-policy evaluation because it evaluated compliance with the declared forgery-risk requirement rather than mutation-test adequacy.

The performance evaluation was conducted at two levels and then composed for deadline-budget analysis. First, SM4-CMAC generation and verification latency was measured directly on the TC397 target platform for authenticated-input lengths of 8, 16, and 32 B. The measurements used the same software cryptographic path deployed by the SecOC prototype, ensuring platform consistency between functional validation and cryptographic timing evaluation. Second, CAN-FD communication timing was evaluated using deterministic fixed-priority non-preemptive worst-case response-time analysis. The two components were first characterized separately to preserve their different timing semantics and were subsequently composed in Section 5.8 to assess whether the evaluated software cryptographic path fitted within the end-to-end deadline budget. ECU scheduling, application processing, and other non-cryptographic software delays remained outside this composed timing-budget check.

The campaign produced 1575 execution records across nine scenarios. Of these, 1535 were message-level execution records, comprising 286 valid and 1249 invalid inputs, while the remaining 40 records corresponded to authenticator-length policy tests. The execution count is reported separately from the number of retained semantic equivalence classes. Repeated executions of the same retained class were used only to assess execution stability or timing variation and were not interpreted as additional semantic test coverage.

The target-platform cryptographic study contained six timing conditions, corresponding to SM4-CMAC generation and verification at 8, 16, and 32 B. For each condition, 30,000 measurements were collected and summarized using the mean, standard deviation, median, P95, P99, and maximum. Bus-level timing was analyzed independently from the periodic CAN-FD traffic configuration. Each message was characterized by its period, relative deadline, priority, release jitter, and worst-case CAN-FD transmission time, while higher-priority interference and non-preemptive blocking were explicitly considered in the response-time analysis. For messages whose relative deadline exceeded their period, all relevant instances within the priority-level busy period were examined when determining the worst-case response time.

The high-load message scenario remained an empirical timing-stress experiment. Its observed application delivery and deadline-miss results are reported separately from the analytical CAN-FD worst-case response time and were not used to parameterize a stochastic queuing model. The analytical response-time result therefore characterized bus-level schedulability under the specified periodic traffic configuration, whereas the high-load experiment captured the end-to-end behavior of the implemented ECU and SecOC processing path.

Before each message-level vector, the controller restored the designated condition and configuration. Authentication, freshness, application delivery, resynchronization action, state update, execution status, and processing latency were recorded after execution to limit state coupling between test sequences.

### 5.2. Experimental Environment and Parameters

The evaluation used the same TC397-based prototype for both SecOC functional testing and cryptographic timing measurements. The target platform was an Infineon KIT_A2G_TC397_5V_TRB_S equipped with a TC397XE device. SM4-CMAC was executed through the deployed software path SecOC → Csm → CryIf → Crypto Driver on TriCore CPU0 operating at 300 MHz. The benchmark task was pinned to CPU0, and the CPU clock was held by the configured PLL during the measurements. Timing was collected using CPU0 STM0 operating at 100 MHz, corresponding to a 10 ns timer resolution. The reported values are raw request-to-completion latencies; the timer-read overhead was not subtracted. The cryptographic context was fixed across all input lengths, with CsmJobId=17, KeyRef=0x1001, and a 128-bit key resident in CPU0 Data Scratch-Pad RAM (DSPR). The implementation was compiled using TASKING VX-toolset for TriCore v6.3r1p10 with -O2 optimization.

For each SM4-CMAC generation and verification condition, 30,000 measurements were collected as five batches of 6000 samples. Each batch was preceded by 200 warm-up executions that are excluded from the reported statistics. Authenticated-input lengths of 8, 16, and 32 B were evaluated. The measurement boundary covered the deployed cryptographic-service request-to-completion path and therefore represents target-platform cryptographic-service latency rather than the execution time of an isolated SM4 round function. The evaluated configuration used the software Crypto Driver and did not enable Hardware Security Module (HSM) offload. Therefore, the reported timing results characterize the deployed software cryptographic path on the TC397 platform and were not interpreted as HSM-backed execution latency. The experimental environment and principal parameters are summarized in Table 4.

Figure 3 shows the prototype used for message-level security and configuration testing. The controller generated valid or anomalous messages from the selected vector and loaded the associated precondition and configuration. The receiver then performed freshness reconstruction, authenticator verification, state updating, and application-delivery control. The same TC397 target platform was also instrumented for SM4-CMAC timing, so the cryptographic measurements reported in Section 5.8 were obtained from the deployed automotive microcontroller unit (MCU) rather than from a separate host platform.

### 5.3. Messages and Test Scenarios

Five anonymized in-vehicle sensing and control messages were selected to cover different periods, end-to-end deadlines, and application semantics. Table 5 lists their periods, deadlines, payload lengths, and authenticated regions. The authenticated region did not always cover the complete payload. This arrangement tested both the blocking of perturbations inside the authenticated region and the consistency between safety-critical fields and the authentication configuration.

Table 6 gives the scenario and sample distribution. Payload tampering and authenticator forgery assessed authenticator verification and post-failure delivery gating. Historical replay, window-boundary inputs, and freshness-value rollover assessed freshness-state handling and its boundaries. Cryptographic-context mapping tested isolation across messages, directions, or security domains. High-load injection jointly examined security gating and real-time availability. Authenticator-length testing checked whether the configured truncation length satisfied the declared attack-attempt budget and risk threshold.

Vectors within one scenario were not distinguished by their final receiver outcome. They were distinguished by the input semantics represented in Equation (20). Across the campaign, the message identity varied among PDU-A–PDU-E where compatible with the scenario. Payload tampering varied the authenticated byte/bit location and perturbation value; authenticator-forgery vectors varied the forged tag location or value pattern; replay vectors varied the historical frame and freshness distance from the receiver checkpoint; window-boundary vectors varied the configured window and the candidate’s position relative to that boundary; rollover vectors varied the pre-wrap freshness checkpoint and low-value rollover candidate; cross-context vectors varied the source–destination, direction, or security-domain context pair; and high-load vectors varied the message, validity class, and traffic profile. These distinctions were retained by the equivalence key even when several vectors subsequently produced the same receiver-response tuple.

### 5.4. Evaluation Measures

IAR measured message-level security. A valid input that was not delivered contributed to FRR; a delivered valid input that exceeded its deadline contributed to DMR. IR recorded missing logs, unconfirmed preconditions, or timestamp-correlation failures. Rejection, authenticated resynchronization, execution errors, and timeouts remained separate response categories rather than being merged into a generic detection result. Authenticator-length findings were reported through NPV, while systematic configuration-mutation adequacy was reported through $MS_{\mathrm{cfg}}$ and the corresponding dimension-specific mutation scores.

Cryptographic timing was summarized by the mean, standard deviation, median, P95, P99, and maximum. CAN-FD communication timing was evaluated using fixed-priority non-preemptive worst-case response-time analysis. For message $m_{i}$, we let $R_{i}^{\mathrm{WCRT}}$ denote the worst-case response time obtained from the periodic traffic configuration. The bus-level residual budget was $S_{i}=D_{i}-R_{i}^{\mathrm{WCRT}}$. Section 5.8 compares this residual budget with the measured sender-side SM4-CMAC generation and receiver-side verification latency. A negative composed headroom indicates that these measured cryptographic costs together with the analytical bus-response bound already exceeded the message deadline, even before other ECU software delays were included. The empirical DMR is still reported separately because it reflects the complete implemented execution path. For zero-event scenarios, the Wilson 95% upper bound was computed using Equation (60).

### 5.5. Message-Level Security and Oracle-Path Results

#### 5.5.1. Overall Results

Table 7 summarizes IAR, FRR, DMR, and IR by scenario. All 160 baseline valid messages passed authentication and freshness checking and were delivered on time. Payload tampering, authenticator forgery, historical replay, window-boundary inputs, freshness-value rollover, and cryptographic-context mapping contributed 1120 invalid inputs; the high-load scenario contributed another 129. None reached the application. All 126 valid high-load inputs were eventually delivered, but 6 exceeded their deadlines. FRR was therefore zero, DMR was 4.76%, and IR was zero for every message-level scenario.

High load caused neither fail-open behavior nor rejection of a valid message by the SecOC security mechanism. All 126 valid high-load inputs were eventually delivered, while 6 exceeded their end-to-end deadlines, yielding a DMR of 4.76% and a Wilson 95% interval of 2.20–10.00%.

The six late records were identified from the archived execution records and synchronized timing traces. For each record, the end-to-end latency $\tau_{j}$ was determined from the corresponding sender-side message-release timestamp and application-delivery timestamp, and the deadline overrun was calculated as $\Delta_{j}=\tau_{j}-D\left(v_{j}\right)$. The PDU identity was obtained from the VectorID and its associated message metadata.

Specifically, three late records belonged to PDU-C, with measured end-to-end latencies of 11.6112, 12.2916, and 13.6094 ms, corresponding to deadline overruns of 1.6112, 2.2916, and 3.6094 ms, respectively. The remaining three late records belonged to PDU-D, with measured end-to-end latencies of 12.0362, 12.7166, and 14.0344 ms, corresponding to overruns of 2.0362, 2.7166, and 4.0344 ms, respectively. Both PDU-C and PDU-D had a 10 ms end-to-end deadline.

The available timing evidence identifies the affected PDU and the end-to-end deadline overrun for each late record, but it does not provide sufficiently fine-grained stage timestamps to uniquely decompose an individual miss into sender-side SecOC processing, CAN-FD arbitration, receiver-side SecOC processing, and application scheduling. We therefore report the message identity and overrun magnitude without assigning a single internal cause to these events. Section 5.8 further evaluates the timing sensitivity of PDU-C and PDU-D through the combined cryptographic and CAN-FD timing-budget analysis.

#### 5.5.2. Multidimensional Responses and Defensive Paths

Table 8 reports authentication, freshness, application delivery, state-update behavior, resynchronization, and execution outcomes. Authentication and freshness describe internal SecOC decisions, whereas application delivery confirms whether the security boundary was enforced. The state-update field further distinguishes ordinary rejection from authenticated freshness advancement and from any unauthenticated update. Rejection and authenticated resynchronization were not mutually exclusive: a rollover candidate could remain blocked while the receiver initiated recovery.

Table 8 aggregates executions by observed receiver-response signature and therefore intentionally hides the input-side variation reported in Table 6. Identical entries within one row do not imply that the corresponding inputs belong to the same semantic equivalence class. For example, payload-tampering vectors may have targeted different authenticated locations and messages while still being correctly blocked through the same authentication-failure path.

Across the 1535 message-level execution records, the observed responses collapsed to six response/timing signatures: 280 valid and on-time deliveries, 6 valid but late deliveries, 749 authentication-failure blocks, 220 freshness-failure blocks, 60 joint authentication/freshness rejections, and 220 authenticated rollover-gated recoveries. These six output signatures characterize the receiver behaviors observed in the campaign; they were not used as the input equivalence relation for test-vector reduction.

The authenticator-length policy checks were configuration-level evaluations and did not share the message-response semantics of authentication, freshness checking, and application delivery. They are therefore excluded from Table 8 and reported in Section 5.6. Across all the executed message-level vectors, no case produced $u_{\mathrm{state}}=\mathrm{UPDATED}\text{\_}\mathrm{UNAUTH}$, which is consistent with the zero IAR observed for every invalid-message scenario.

Payload tampering and authenticator forgery were blocked by the authentication path. All 400 anomalous inputs produced FAIL for authentication, PASS for freshness, no application delivery, and no state update. Because the freshness state followed the baseline sequence, the observations isolated modifications within the authenticated region and forgery of the authenticator field. The corresponding VectorID and authenticated-region configuration provided a reproducible basis for localization.

Historical replay produced a different path. Authentication passed for all 220 historical inputs, but freshness failed, every input was rejected, and the freshness state remained unchanged in all cases. A recorded Secured I-PDU may retain a valid authenticator; replay detection must therefore combine the MAC result with $FV_{\mathrm{last}}$, candidate reconstruction, and the acceptance window. The multidimensional response separates this path from an ordinary authentication failure.

In the rollover scenario, authentication and freshness passed for all 220 inputs, but none was delivered, all triggered authenticated resynchronization, and all 220 cases recorded $u_{\mathrm{state}}=\mathrm{UPDATED}\text{\_}\mathrm{AUTH}$. Rollover security therefore depends on cycle-transition gating and application-delivery policy, not only on the local return values of the authentication and freshness interfaces. A low-value candidate can be parsed and locally verified, yet the receiver must keep the delivery gate closed until the new cycle has an authenticated basis.

For the cryptographic-context scenario, freshness passed for all 220 cross-context inputs, whereas authentication failed, every message was rejected, and the receiver freshness state remained unchanged. The baseline mapping therefore isolated messages, directions, and security domains through the assigned key or cryptographic job. For the 60 window-boundary inputs, both authentication and freshness returned FAIL, and no input was delivered. This is reported as an implementation-specific joint-rejection path rather than being attributed solely to freshness-window enforcement; the freshness failure still satisfied the mechanism evidence required by Equation (35).

Under high load, authentication failed for all 129 invalid inputs and passed for all 126 valid inputs; freshness passed for every input. The 129 invalid inputs left the freshness state unchanged, whereas the 126 valid inputs advanced it with authenticated updates. No invalid input reached the application, so load did not weaken delivery gating after authentication failure. All valid inputs were eventually delivered, although 6 missed their deadlines. IAR and FRR therefore remained zero, while DMR rose to 4.76%.

#### 5.5.3. Binary-Oracle Ablation

To directly test the diagnostic value of the multidimensional oracle, we re-scored all 1535 message-level execution records using the binary oracle in Equation (49). Because the evidence was complete for all message-level executions, the binary result contains only two labels: 286 records were classified as ACCEPT and 1249 as REJECT.

Table 9 compares these binary labels with the response/timing signatures retained by CAST-SecOC. The same execution records were used in both cases; only the oracle input was reduced.

The binary oracle preserved the final application-boundary observation, but it collapsed the six response/timing signatures into two outcomes. In particular, the 749 authentication-failure blocks, 220 freshness-failure blocks, 60 joint authentication/freshness rejections, and 220 authenticated rollover-gated recoveries all became REJECT. Thus, a binary result could not determine whether a message was blocked by authentication, freshness checking, a joint rejection path, or an authenticated rollover-recovery procedure.

The loss is also visible for valid traffic. The six late valid messages were classified as ACCEPT together with the 280 on-time deliveries, so the binary oracle did not expose the deadline violations. More generally, an unauthenticated freshness-state update that remains blocked at the application boundary would also appear as REJECT and would not be identified from $d_{\mathrm{app}}$ alone. Similarly, a binary rejection cannot verify whether rollover recovery was based on authenticated resynchronization evidence.

The comparison therefore does not show a difference in final application-boundary counts: both interpretations observed that all 1249 invalid inputs were blocked and all 286 valid inputs were delivered. The additional value of CAST-SecOC lies in diagnosis rather than in changing those final counts. By retaining authentication, freshness, recovery, state-update, execution, and timing evidence, the multidimensional oracle distinguishes mechanism paths and identifies failure modes that an application-only binary observation cannot attribute.

#### 5.5.4. Statistical Interpretation Under Finite Samples

Table 10 reports the one-sided 95% Wilson upper confidence bounds for the zero-event scenarios. The 400 direct authentication anomalies yielded an upper bound of 0.67%, whereas the 60 window-boundary inputs yielded 4.32%. These bounds characterize the evaluated sample sizes within each scenario and are not interpreted as system-wide failure probabilities.

### 5.6. Configuration-Fault Localization

#### 5.6.1. Authenticator-Length Policy

The authenticator-length policy uses an upper bound of q=1000 attack attempts and a maximum permitted random-forgery probability of $\theta =2^{-32}$. Applying Equation (45), Table 11 reports the policy verdicts for the four evaluated authenticator lengths. The 16-, 24-, and 32-bit configurations exceeded the threshold; the 64-bit configuration satisfied the policy.

Among the 40 authenticator-length policy execution records, 20 corresponded to policy-violating configurations that were accepted for loading or execution; hence NPV=20. The remaining 20 records did not simultaneously satisfy both conditions. This result identifies a policy-enforcement gap and does not represent successful online forgery attempts. Each accepted policy-violation record retains its ConfigID and policy basis.

#### 5.6.2. Systematic Configuration-Mutation Results

The systematic mutation campaign generated 24 first-order configuration mutants from the nine dimensions in Equation (16). Mutant generation was independent of the defect criteria in Section 4.5.9, and the same fixed regression-vector set was executed against all applicable mutants.

Among the 24 generated mutants, two were rejected during configuration loading and two were classified as equivalent to another generated mutant under the observable configuration semantics. The remaining 20 mutants were executable and non-equivalent and therefore formed the denominator of the mutation score. Table 12 summarizes the results by configuration dimension.

Of the 20 executable non-equivalent mutants, 16 were killed by the fixed regression suite and four survived. The resulting configuration mutation score was

$$MS_{\mathrm{cfg}}=\frac{16}{20}=80.00\%.$$

The results differ substantially across configuration dimensions. All executable mutants involving authenticator length *ℓ*, acceptance-window size *W*, rollover/resynchronization policy ρ, authenticated-region configuration *A*, and cryptographic-context mapping κ were killed, yielding a dimension-specific mutation score of 100% for these dimensions. In contrast, one mutant survived in each of the λ, *b*, $\pi^{\mathrm{timeout}}$, and $\pi^{\mathrm{persist}}$ dimensions, for which $MS_{d}=50.00\%$.

The surviving λ mutant was not distinguished because the fixed freshness sequences did not reach a reconstruction boundary at which the altered transmitted freshness-field length changed the oracle-visible behavior. The surviving *b* mutant similarly required execution near a full freshness-counter boundary that was not reached by the existing replay and rollover sequences. The surviving $\pi^{\mathrm{timeout}}$ mutant did not activate a distinct timeout path within the duration of the current regression vectors, while the surviving $\pi^{\mathrm{persist}}$ mutant required a reset or restart sequence for its persistence behavior to become observable.

These surviving mutants are retained as explicit adequacy findings rather than being removed from the analysis. They show that exercising a configuration dimension does not necessarily imply behavioral sensitivity to every configuration change within that dimension. Accordingly, the 80.00% mutation score is interpreted as an empirical measure of the current regression suite’s configuration-fault detection capability rather than as evidence of exhaustive fault coverage.

### 5.7. Dimensional Coverage and Mutation-Based Test Adequacy

The execution count was not used as a semantic-coverage metric. Structural coverage was computed from covered obligations and retained equivalence classes, whereas repeated executions of the same class contributed only to empirical outcome and timing statistics. Consequently, increasing the number of repeated executions did not increase the reported dimensional coverage.

Dimensional coverage and mutation-based adequacy characterize different properties of the test campaign and are therefore reported separately. Dimensional coverage indicates whether a receiver condition, freshness relation, configuration boundary, perturbation operator, or requirement class was exercised. It does not by itself demonstrate that every behavior associated with that dimension could be distinguished.

Table 13 reports structural test coverage, whereas Table 12 reports mutation-based behavioral fault-detection adequacy. Although the test campaign exercised the intended configuration dimensions, the mutation study achieved ${\mathrm{MS}}_{\mathrm{cfg}}=80.00\%$, with four surviving mutants in λ, *b*, $\pi^{\mathrm{timeout}}$, and $\pi^{\mathrm{persist}}$. These surviving cases demonstrate that configuration coverage and configuration-fault sensitivity should not be interpreted as equivalent notions.

The synchronized condition supported valid communication, payload tampering, authenticator forgery, historical replay, out-of-window inputs, cross-context inputs, and high-load tests. The rollover-guard condition supported cycle-transition and delivery-gating tests near the upper bound of the full freshness value. Window-boundary and rollover cases were represented by freshness relations rather than perturbation operators; authenticator length, acceptance-window size, and cryptographic-context mapping were configuration dimensions. Each covered obligation was linked to its VectorID, ConfigID, and execution trace. The two executed conditions characterize the observable preconditions used in this study and are not claimed to exhaust the internal state space of a Freshness Manager.

### 5.8. Target-Platform Cryptographic Latency and CAN-FD Response-Time Analysis

The cryptographic timing assessment was conducted directly on the TC397 target MCU used by the SecOC prototype. Table 14 reports SM4-CMAC generation and verification latency for authenticated inputs of 8, 16, and 32 B. All six conditions used the same CPU, compiler, cryptographic context, key, and deployed software path; only the authenticated-input length and operation type were varied.

CAN-FD communication timing was evaluated using deterministic fixed-priority non-preemptive response-time analysis. The analysis used the individual periods, deadlines, priorities, transmission times, and release jitter of the periodic foreground and background messages rather than an aggregate stochastic queuing model. Cryptographic-service latency and CAN-FD response time are first characterized separately because they represent different components of the execution path. They are then composed below as a partial end-to-end timing-budget check by comparing the measured sender-side generation and receiver-side verification costs with the residual budget left by the analytical CAN-FD Worst-Case Response Time (WCRT). Non-cryptographic ECU and application delays remain outside this composed model.

#### 5.8.1. TC397 Target-Platform SM4-CMAC Latency

Table 14 shows that both SM4-CMAC generation and verification latency increased with authenticated-input length on the TC397 platform. For generation, the mean latency increased from 1.5806 ms at 8 B to 1.9211 ms at 16 B and 2.5797 ms at 32 B. The corresponding verification means were 1.6210, 1.9609, and 2.6201 ms, respectively. Increasing the authenticated input from 16 to 32 B increased the mean generation latency by 34.28% and the mean verification latency by 33.62%.

The percentile results followed the same trend. Generation P99 increased from 1.7544 ms at 8 B to 2.1344 ms at 16 B and 2.8574 ms at 32 B. Verification P99 increased from 1.8015 ms to 2.1797 ms and 2.9052 ms over the same input lengths. From 16 to 32 B, the P99 increases were 33.87% for generation and 33.28% for verification. Verification remained slightly slower than generation across all three input lengths, with the difference in mean latency remaining below approximately 2.6%.

The mean and median values were close under all six conditions, while the standard deviations ranged from 0.0748 to 0.1207 ms. The maximum observed latency remained below 3.68 ms. The gap between P99 and the maximum indicates the presence of occasional tail-latency events, but the overall increase with authenticated-input length was consistent across the mean, P95, and P99 statistics. These measurements therefore characterize the request-to-completion latency of the deployed TC397 software cryptographic-service path rather than the execution time of an isolated SM4 primitive.

#### 5.8.2. CAN-FD Worst-Case Response-Time Analysis

The evaluated communication workload consisted of periodic SecOC messages sharing a priority-arbitrated CAN-FD bus. The five foreground messages had periods of 5, 10, or 100 ms, and CAN-FD frame transmission was non-preemptive once a frame won arbitration. Accordingly, the bus timing analysis followed fixed-priority non-preemptive response-time analysis rather than an M/M/1 approximation.

The analysis followed the CAN response-time framework introduced by Tindell et al. [32] and the revised formulation of Davis et al. [33]. The latter requires all relevant message instances within a priority-level busy period to be examined when determining the worst-case response time. This is particularly important for PDU-C and PDU-D, whose periods are 5 ms while their relative deadlines are 10 ms.

For each message $m_{i}$, we let $T_{i}$ denote its period, $D_{i}$ its relative deadline, $J_{i}$ its release jitter, and $C_{i}^{\mathrm{FD}}$ its worst-case CAN-FD transmission time. We let hp(i) and lp(i) denote the sets of messages with higher and lower priority than $m_{i}$, respectively. The maximum non-preemptive blocking time was

(63) $$B_{i}=\max_{k\in \mathrm{lp}(i)}C_{k}^{\mathrm{FD}}.$$

We let hep(i)=hp(i)∪{i}. The priority-level busy period was obtained by fixed-point iteration:

(64) $$t_{i}^{\left(n+1\right)}=B_{i}+\sum_{k\in \mathrm{hep}(i)}\left\lceil \frac{t_{i}^{\left(n\right)}+J_{k}}{T_{k}}\right\rceil C_{k}^{\mathrm{FD}},\;t_{i}^{\left(0\right)}=C_{i}^{\mathrm{FD}}.$$

The number of instances of $m_{i}$ released before the end of the busy period was

(65) $$Q_{i}=\left\lceil \frac{t_{i}+J_{i}}{T_{i}}\right\rceil .$$

For the *q*-th instance, where $q=0,\ldots ,Q_{i}-1$, the queuing delay was computed iteratively as

(66) $$w_{i}^{\left(n+1\right)}\left(q\right)=B_{i}+qC_{i}^{\mathrm{FD}}+\sum_{k\in \mathrm{hp}(i)}\left\lceil \frac{w_{i}^{\left(n\right)}\left(q\right)+J_{k}+\tau_{\mathrm{bit}}}{T_{k}}\right\rceil C_{k}^{\mathrm{FD}},$$

where $\tau_{\mathrm{bit}}$ denoted one arbitration-phase bit time. The response time of the *q*-th instance was

(67) $$R_{i}\left(q\right)=J_{i}+w_{i}\left(q\right)-qT_{i}+C_{i}^{\mathrm{FD}},$$

and the worst-case response time of message $m_{i}$ was

(68) $$R_{i}^{\mathrm{WCRT}}=\max_{0\leq q<Q_{i}}R_{i}\left(q\right).$$

The value $C_{i}^{\mathrm{FD}}$ was calculated from the actual secured CAN-FD frame configuration, including the arbitration-phase and data-phase portions of the frame, using the configured 500 kbit/s arbitration bitrate and 2 Mbit/s data-phase bitrate. Background traffic was represented by its individual period, priority, release jitter, and frame transmission time; aggregate bus utilization alone was not used as an input to the response-time recurrence.

#### 5.8.3. Message-Level WCRT and Remaining Timing Budget

Table 15 reports the message-level CAN-FD worst-case response-time results under the 30%, 85%, and 95% traffic configurations. For each message, the analysis considered its period $T_{i}$, relative deadline $D_{i}$, worst-case CAN-FD transmission time $C_{i}^{\mathrm{FD}}$, non-preemptive blocking term $B_{i}$, the number of message instances $Q_{i}$ examined within the priority-level busy period, and the resulting worst-case response time $R_{i}^{\mathrm{WCRT}}$. The remaining timing budget was defined as

(69) $$S_{i}=D_{i}-R_{i}^{\mathrm{WCRT}},$$

where $S_{i}$ denotes the portion of the message deadline remaining after the analytical CAN-FD response-time bound.

The calculated response times increased consistently as the traffic load rose. At 95% load, the WCRT values of PDU-A, PDU-B, and PDU-E were 9.2596, 9.4776, and 9.7016 ms, respectively. Their longer deadlines left bus-level residual budgets of 10.7404, 10.5224, and 190.2984 ms.

PDU-C and PDU-D were more timing-constrained. For PDU-C, $R_{i}^{\mathrm{WCRT}}$ increased from 1.9096 ms at 30% load to 7.4096 ms at 85% load and 8.4096 ms at 95% load. Its residual budget consequently decreased from 8.0904 to 2.5904 and 1.5904 ms. For PDU-D, the corresponding WCRT values were 2.1146, 7.8346, and 8.8346 ms, leaving 7.8854, 2.1654, and 1.1654 ms, respectively. PDU-D therefore had the smallest bus-level residual budget under the 95% traffic configuration.

The multiple-instance analysis also became relevant for the two short-period control messages. For PDU-C and PDU-D, $Q_{i}=1$ at 30% load but increased to $Q_{i}=2$ at both 85% and 95% load. For PDU-A, PDU-B, and PDU-E, $Q_{i}$ remained equal to one under all three traffic configurations.

The transmission-time term $C_{i}^{\mathrm{FD}}$ and the blocking term $B_{i}$ remained unchanged for a given PDU. The increase in $R_{i}^{\mathrm{WCRT}}$ therefore resulted from the additional higher-priority interference introduced by the denser traffic configuration. The response-time behavior was consequently message-specific rather than a function of aggregate utilization alone.

This result is particularly relevant to PDU-C and PDU-D. PDU-C carries motor-speed and actual-torque feedback, while PDU-D carries commanded torque and drive-enable information. Both use a 5 ms period and a 10 ms end-to-end deadline. The response-time analysis therefore identified these two control messages as the most timing-sensitive traffic in the evaluated high-load configuration. The NORMAL/HIGH labels refer to execution-condition classes used in the message-level campaign, whereas 30%, 85%, and 95% denote the separate periodic traffic configurations used in the analytical CAN-FD response-time analysis.

The residual budgets in Table 15 can be directly compared with the target-platform cryptographic-service latencies in Table 14. For an authenticated-input length *n*, the measured mean cryptographic cost of one authenticated transmission was

(70) $$L_{\mathrm{crypto}}^{\mathrm{mean}}\left(n\right)=L_{\mathrm{gen}}^{\mathrm{mean}}\left(n\right)+L_{\mathrm{ver}}^{\mathrm{mean}}\left(n\right).$$

The resulting values were 3.2016, 3.8820, and 5.1998 ms for 8, 16, and 32 B, respectively. Comparing these costs with the bus-level residual budget $S_{i}=D_{i}-R_{i}^{\mathrm{WCRT}}$ gave the composed timing headroom

(71) $$H_{i}\left(n\right)=S_{i}-L_{\mathrm{crypto}}^{\mathrm{mean}}\left(n\right).$$

A negative $H_{i}\left(n\right)$ indicates that the analytical CAN-FD WCRT together with the measured sender-side generation and receiver-side verification cost already exceeded the message deadline, even before ECU scheduling, application processing, and other non-cryptographic software delays were included.

For PDU-C, the residual budget was 8.0904 ms at 30% load, 2.5904 ms at 85% load, and 1.5904 ms at 95% load. For PDU-D, the corresponding values were 7.8854, 2.1654, and 1.1654 ms. At 30% load, both messages retained positive headroom for all three measured authenticated-input lengths. In contrast, at 85% load even the shortest 8 B condition exceeded the residual budget: the composed mean headroom was −0.6112 ms for PDU-C and −1.0362 ms for PDU-D. At 95% load, these deficits increased to −1.6112 and −2.0362 ms, respectively, and the deficits were larger for the 16 and 32 B conditions.

Equivalently, for PDU-D at 95% load, the analytical bus response time of 8.8346 ms combined with the 8 B mean SM4-CMAC generation and verification latencies yielded 8.8346+1.5806+1.6210=12.0362 ms, exceeding the 10 ms deadline by 2.0362 ms. The same conclusion held for PDU-C and PDU-D at both 85% and 95% load for all three measured input lengths, whereas both messages fitted within the composed budget at 30% load. For PDU-D at 95% load, the combined generation-and-verification latency would need to decrease from 3.2016 ms to at most 1.1654 ms even for the 8 B condition, a reduction of at least 63.6% before other software delays could be considered. This quantifies the timing motivation for HSM offload or equivalent cryptographic acceleration under high bus load.

Using the separately measured stage-wise P99 generation and verification latencies further widened the deficit. Their sums were 3.5559, 4.3141, and 5.7626 ms for 8, 16, and 32 B, respectively, and remained above the residual budgets of PDU-C and PDU-D at both 85% and 95% load. These P99 sums were used as a conservative component-wise budget comparison and were not interpreted as the empirical P99 of the complete end-to-end latency distribution.

The prototype high-load experiment remained a separate end-to-end observation: all 126 valid high-load messages were eventually delivered, while 6 exceeded their application deadlines, corresponding to an empirical DMR of 4.76%. The composition above explains quantitatively why the short-period control traffic had little or negative timing headroom under high load, but it does not assign an individual empirical miss to a specific internal processing stage.

### 5.9. Answers to the Research Questions and Discussion

RQ1—Executable and traceable test-vector construction. The campaign produced 1575 execution records from 315 retained semantic equivalence classes, including 307 message-level classes and 8 authenticator-length policy classes. Each retained class was executed five times, with repeated runs used for execution stability rather than additional semantic coverage. The 1535 message-level records produced six observed response/timing signatures. This separates input diversity, execution repetition, and observed receiver behavior while preserving traceability through VectorID, ConfigID, bus traces, ECU logs, and verdicts.

RQ2—Diagnostic value beyond a binary oracle. Re-scoring the 1535 message-level records with an application-only binary oracle produced 286 ACCEPT and 1249 REJECT outcomes. The same records formed six response/timing signatures under CAST-SecOC. A binary REJECT merged authentication failure, freshness failure, joint authentication/freshness rejection, and authenticated rollover-gated recovery, while the six late valid deliveries were merged with the 280 on-time ACCEPT outcomes.

The comparison shows that the multidimensional oracle does not change the final application-level accept/reject counts. Its value lies in retaining the mechanism and timing evidence needed to distinguish blocking and recovery paths, state-update basis, and deadline violations. The configuration-mutation campaign provided a separate adequacy result: 16 of 20 executable non-equivalent mutants were killed, yielding ${\mathrm{MS}}_{\mathrm{cfg}}=80.00\%$.

RQ3—Target-platform cryptographic latency and composed timing headroom. Direct measurements on the TC397 show that SM4-CMAC latency increased with authenticated-input length. Increasing the input from 16 to 32 B raised the mean generation and verification latencies by 34.28% and 33.62%, respectively; the corresponding P99 increases were 33.87% and 33.28%. These values characterize the deployed software cryptographic-service path rather than isolated SM4 primitive execution.

The CAN-FD response-time analysis showed a load-dependent reduction in timing headroom. At 95% load, PDU-D retained only 1.1654 ms after the bus-level WCRT and PDU-C retained 1.5904 ms. In comparison, the measured mean sender-plus-receiver SM4-CMAC cost was already 3.2016 ms for the 8 B condition and increased to 3.8820 and 5.1998 ms for 16 and 32 B. The composed analysis therefore yielded negative timing headroom for both PDU-C and PDU-D at 85% and 95% traffic load for all three evaluated input lengths, whereas the corresponding headroom remained positive at 30% load. In the most constrained case, PDU-D at 95% load would require the combined 8 B cryptographic cost to decrease by at least 63.6% merely to fit within the remaining 10 ms deadline budget before other ECU software overhead could be considered.

Record-level inspection of the archived high-load execution traces showed that all six observed deadline misses occurred in PDU-C and PDU-D, with measured overruns ranging from 1.6112 to 4.0344 ms. The composed analysis in Section 5.8 provides an analytical explanation for why these two short-period messages exhibit the smallest timing headroom under high bus load.

The composition is a timing-budget check rather than a complete end-to-end schedulability guarantee because ECU scheduling, application processing, and other non-cryptographic software delays are not included. The P99-based component-wise comparison further widens the composed timing-budget deficit under high load.

For real-vehicle application, NET vectors represent the transferable on-network attack surface, whereas LAB vectors retain their role as authorized verification procedures for configuration and receiver-state boundary testing rather than field-level adversary capabilities.

## 6. Conclusions

This paper presented CAST-SecOC, a configuration-aware multidimensional testing method for AUTOSAR SecOC over CAN FD. The method combines configuration-aware test-vector construction with a multidimensional oracle that jointly considers authentication, freshness, application delivery, resynchronization, state updates, and timing. The evaluation produced 1575 execution records from 315 semantic equivalence classes. All 1249 invalid messages were blocked, while all 286 valid messages were eventually delivered. Of 126 valid high-load messages, 6 missed their deadlines, with all 6 misses confined to PDU-C and PDU-D. Binary-oracle ablation showed that the same records collapsed to only ACCEPT/REJECT outcomes, whereas CAST-SecOC retained six distinct response/timing signatures. Systematic mutation testing killed 16 of 20 executable non-equivalent configuration mutants, yielding ${\mathrm{MS}}_{\mathrm{cfg}}=80.00\%$. Composed timing-budget analysis further showed that PDU-C and PDU-D did not retain sufficient residual deadline budget at 85% or 95% traffic load to accommodate even the mean combined TC397 software SM4-CMAC generation-and-verification cost for any of the three evaluated input lengths, whereas positive timing headroom remained at 30% load. This result quantifies the motivation for cryptographic acceleration or HSM offload for short-period SecOC control traffic under high bus load.

Future work will extend CAST-SecOC to broader freshness mechanisms and receiver-state conditions, improve test sensitivity to the configuration dimensions associated with surviving mutants, and evaluate additional cryptographic-service implementations and in-vehicle Ethernet scenarios. Chaos-based and memristive-neuron-based pseudo-random parameter generation will also be investigated to increase perturbation diversity for fuzzing and robustness testing. Further real-vehicle studies will assess the transferability of the proposed testing and evidence framework beyond the current prototype configuration.

## Figures and Tables

**Figure 1:**
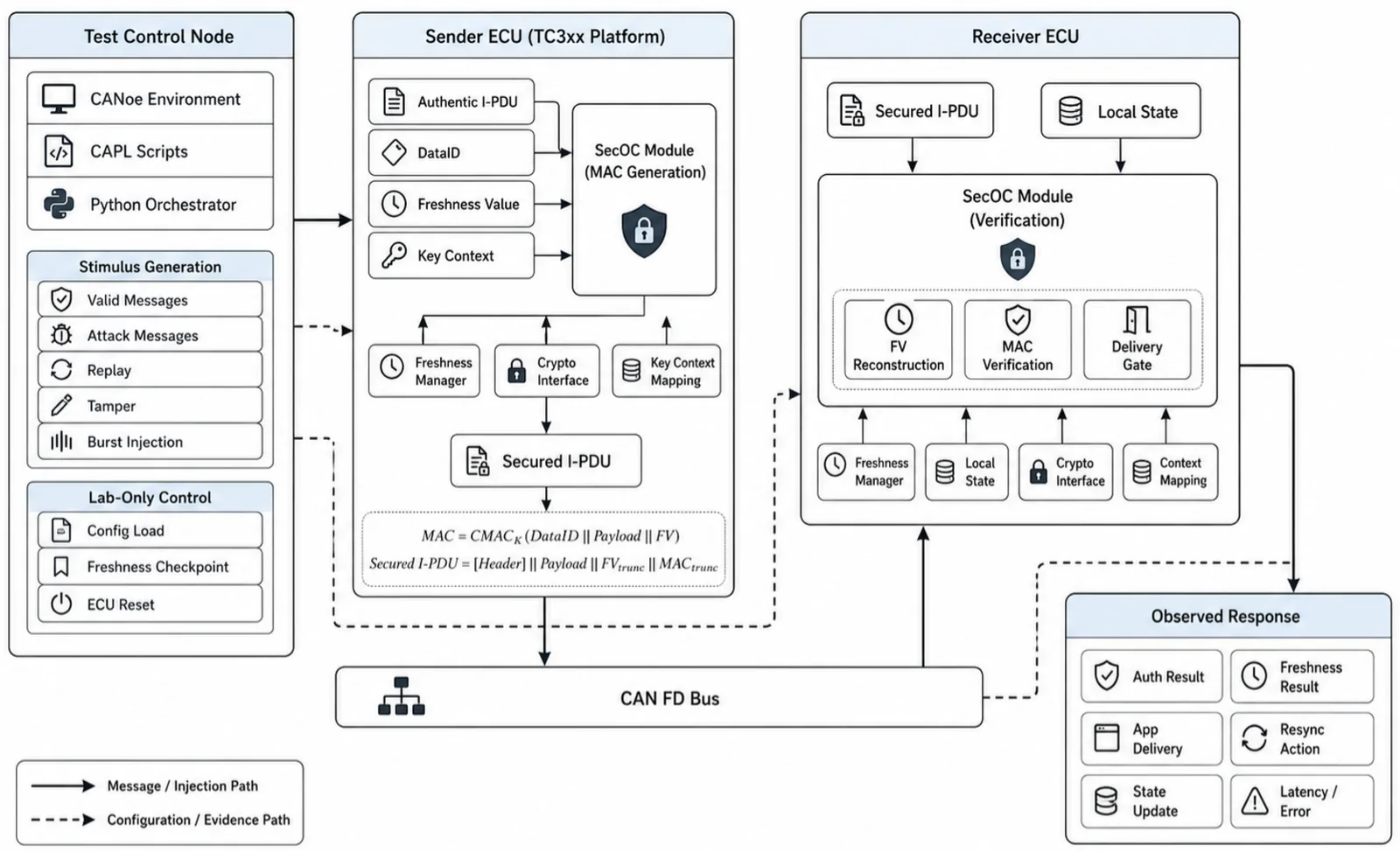
CAST-SecOC system model and evidence flow.

**Figure 2:**
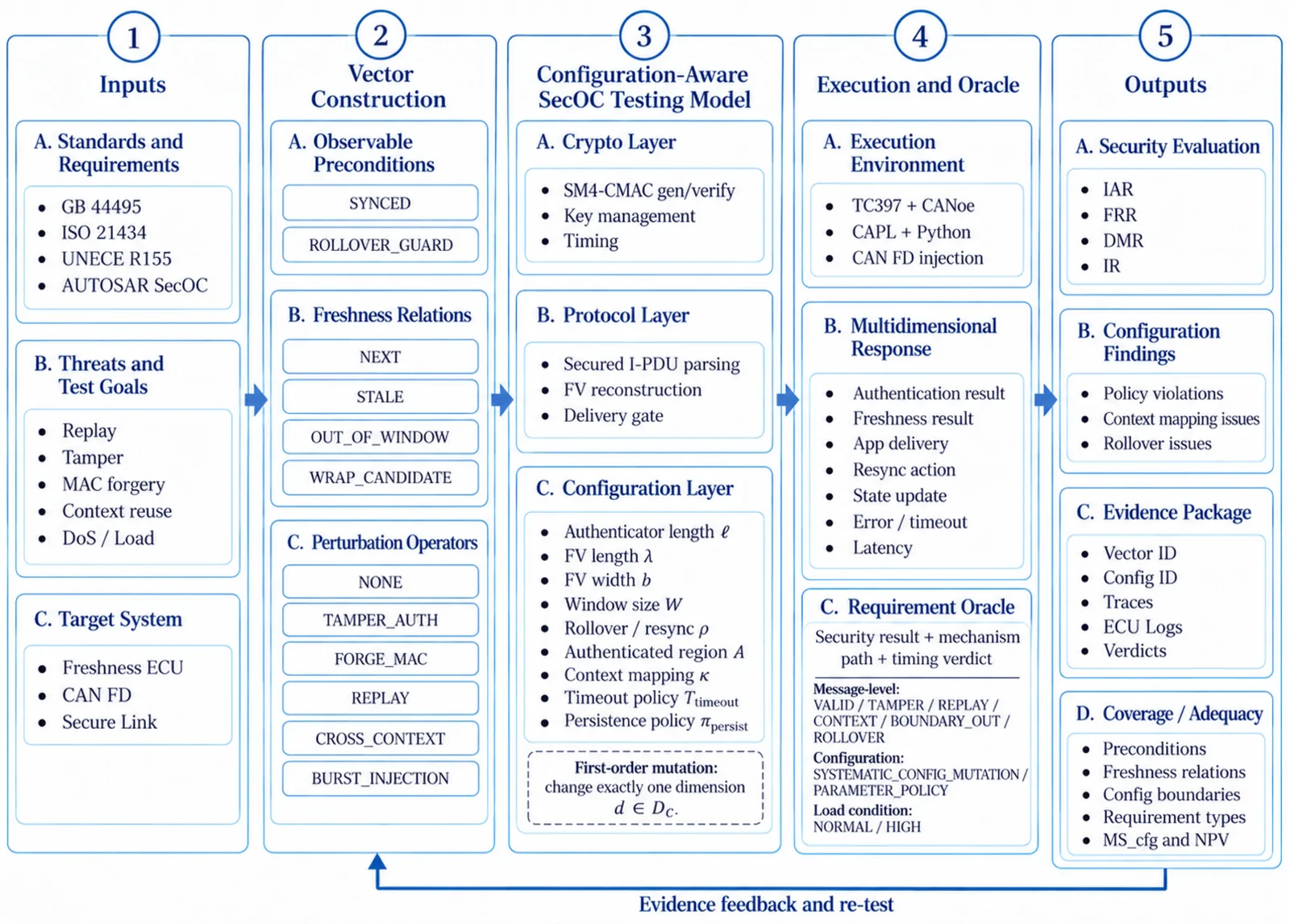
CAST-SecOC configuration-aware multidimensional testing workflow.

**Figure 3:**
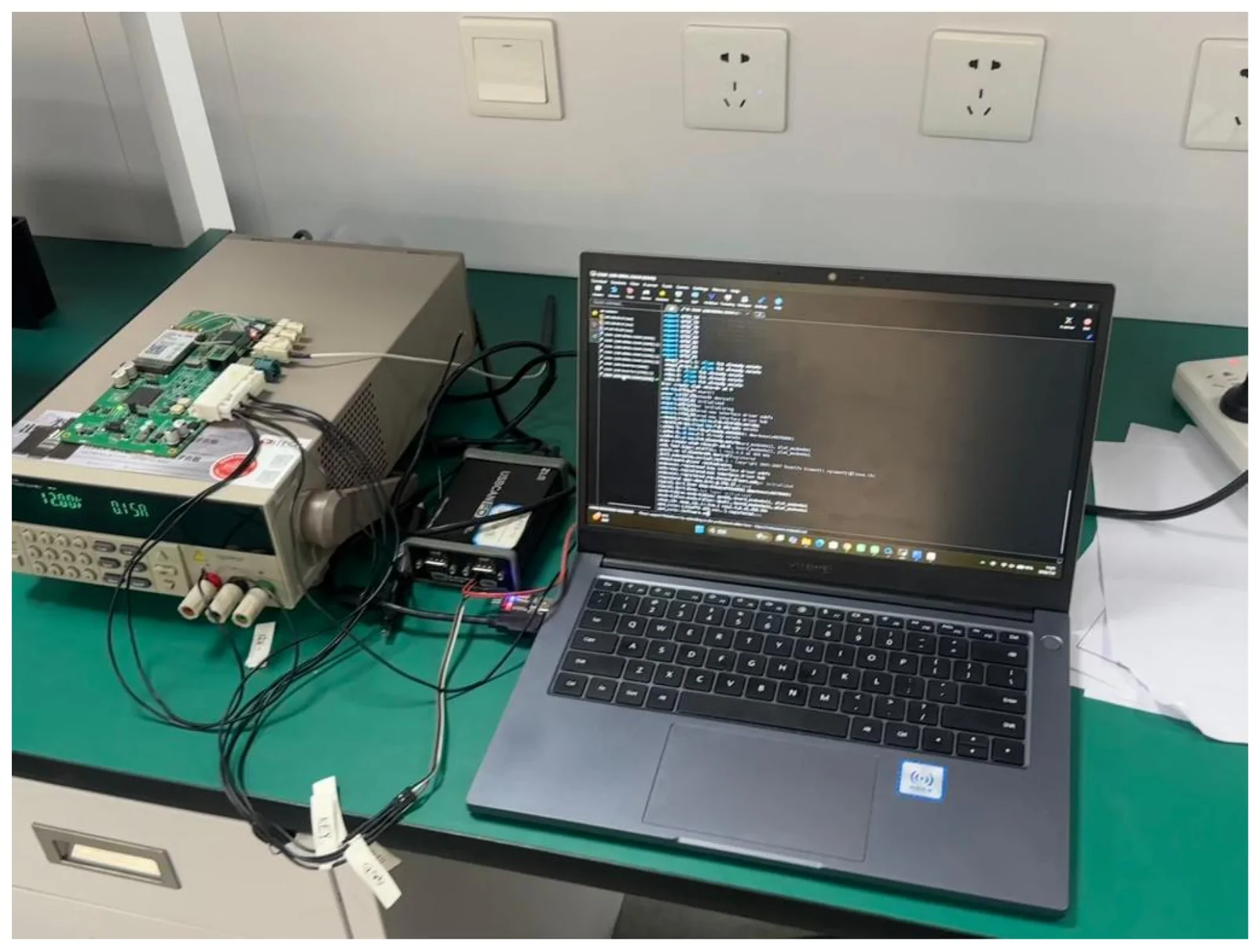
CAST-SecOC prototype-link test platform.

**Table 1:** Defect-oriented comparison with representative recent studies from a SecOC deployment-verification perspective.

Representative Work	Main Objective and Reported Evaluation Scale	State Condition Modeling	Configuration Boundaries	Limitations for SecOC Deployment Verification
CANASTA [11]				
IEEE TVT, 2023	Analyzes the schedulability impact of authentication for CAN, CAN FD, and CAN XL. The evaluation contains two automotive case studies together with randomized schedulability experiments over a broad range of message sets.	0/2. No executable SecOC receiver condition is modeled; the analysis focuses on message response time and schedulability.	0/9. Authentication-related timing and MAC transmission strategies are evaluated, but the nine SecOC deployment- configuration dimensions are not exercised as configuration boundaries.	Provides detailed timing and schedulability analysis, but it does not verify freshness reconstruction, rollover gating, authenticated-region selection, cryptographic-context mapping, or receiver-state updates. It therefore cannot localize a failure to a specific SecOC deployment configuration.
de Andrade et al. [15]				
Computers & Security, 2023	Proposes a CAN-FD security architecture using AES-256 and HMAC/SHA-256. Real-vehicle CAN data are used as inputs, and seven frame/security cases are analyzed in terms of encrypted data size, processing delay, and bus load.	0/2. No explicit SecOC freshness or rollover receiver condition is modeled.	0/9. Payload partitioning and cryptographic parameters are varied for the proposed frame design, but they are not AUTOSAR SecOC configuration boundaries as defined in Equation (16).	Evaluates the design and performance of a protected CAN-FD frame rather than a deployed SecOC instance. Freshness-window behavior, rollover/ resynchronization, state persistence, authenticated-region faults, application-delivery gating, and configuration mutants are not examined.
TrinitySec [13]				
IEEE TDSC, 2024	Develops an integrated authentication, authorization, confidentiality, and integrity framework for CAN-FD. Security properties are analyzed with ProVerif, and performance is evaluated on STM32H743IIT- and STM32G0B1CBT-based hardware platforms in terms of computation, communication, memory, and storage overhead.	0/2. Security-protocol phases are modeled, but not executable SecOC receiver conditions such as SYNCED or ROLLOVER_GUARD.	0/9. Security-framework parameters are evaluated, but SecOC deployment parameters are not systematically varied as configuration boundaries.	Demonstrates the security and efficiency of an integrated CAN-FD protection framework, but it does not generate configuration-constrained SecOC test vectors or jointly observe authentication, freshness, resynchronization, state update, and application-delivery behavior for configuration-fault localization.
CAN-MM [12]				
IEEE TVT, 2024	Introduces frequency- modulated MAC transport to preserve compatibility with legacy CAN while reducing MAC-transmission overhead. Evaluation includes a three-node LTSpice CAN network and a loop-back hardware prototype with a custom CAN-MM transmitter, standard CAN reception, and vehicle-derived noise profiles.	0/2. The evaluation concerns physical-layer and MAC-transport behavior rather than SecOC receiver-state conditions.	0/9. Carrier, modulation, and MAC-transport parameters are investigated, but none of the nine SecOC deployment dimensions is treated as a configuration-verification boundary.	Focuses on MAC carriage, backward compatibility, and physical-layer robustness. It does not cover truncated-freshness reconstruction, acceptance-window boundaries, rollover gating, timeout/persistence policies, or a traceable SecOC configuration-defect oracle.
DBCSec [14]				
IEEE TDSC, 2025	Proposes DBC-guided semi-decentralized key management and secure CAN-FD communication. The prototype uses one Raspberry Pi 3B and three STM32H743IIT6 nodes, with computational cost, communication overhead, and bus-load effects evaluated on CAN/CAN-FD.	0/2. Communication and key-management phases are modeled, but not SecOC freshness-state or rollover receiver conditions.	1/9 ^†^. The DBC-guided communication/key relationship is related to cryptographic-context mapping κ; the remaining SecOC configuration boundaries in Equation (16) are not explicitly verified.	Provides relationship-aware key organization and secure communication, but it does not verify SecOC receiver behavior across freshness, authenticated-region, rollover, timeout, and persistence boundaries, nor provide a requirement-level oracle for configuration-mutant localization.
CAST-SecOC				
This work	Performs configuration-aware deployment verification of AUTOSAR SecOC over CAN FD. The prototype campaign produces 1575 execution records from 315 retained semantic equivalence classes across nine scenarios and five sensing/control PDUs on a TC397-based prototype.	2/2. Both SYNCED and ROLLOVER_GUARD are explicitly prepared, confirmed, exercised, and restored during testing.	9/9. Explicitly models *ℓ*, λ, *b*, *W*, ρ, *A*, κ, $\pi_{\mathrm{timeout}}$, and $\pi_{\mathrm{persist}}$ as SecOC deployment- configuration dimensions.	The current evaluation is limited to two observable receiver conditions, a counter-based freshness configuration, the selected configuration space, and the evaluated TC3xx prototype. It does not claim exhaustive coverage of all internal Freshness Manager states or all production-vehicle configurations.

^†^ The 1/9 entry for DBCSec denotes a functional correspondence: its DBC-guided communication/key relationship is conceptually related to κ, but it is not equivalent to the complete AUTOSAR SecOC AuthServiceConfigRef/CsmJobId/KeyRef cryptographic-context configuration used in CAST-SecOC.

**Table 2:** Notations.

Symbol	Meaning	Symbol	Meaning
$m_{i}$	The *i*-th message under test	$c_{r}$	The *r*-th SecOC configuration
$P_{\mathrm{auth}}$	Authentic I-PDU	$P_{\mathrm{wire}}$	On-wire Secured I-PDU
A=(o,n)	Authenticated-region parameters	I	Authenticated byte-index set
*ℓ*	Truncated authenticator length	λ	Transmitted freshness-field length
*b*	Full freshness-value width	*W*	Acceptance window
$S_{O}$	Observable receiver-condition set	$R_{FV}$	Input freshness-relation set
κ	Internal cryptographic-context mapping	$z_{\mathrm{exec}}$	Execution status
$d_{\mathrm{app}}$	Application-delivery indicator	$a_{\mathrm{resync}}$	Resynchronization-action type
D(v)	Deadline associated with vector *v*	O(v,r)	Requirement-level oracle

**Table 3:** Requirement-level oracle for each message-level test-vector class.

Vector Class	Application Outcome	Expected Mechanism Path	State Update	Principal Failure Condition
Valid message	Must be delivered	Authentication and freshness both pass	Authenticated update permitted	No delivery or abnormal verification result; late delivery is counted separately in DMR
Payload or authenticator tampering	Must not be delivered	Authentication fails while freshness remains valid	No unauthenticated update	Invalid delivery or rejection by an unrelated earlier mechanism
Historical replay	Must not be delivered	Authentication passes and freshness fails	No rollback	Historical message delivered or receiver state rolled back
Cross-context input	Must not be delivered	Freshness remains valid and authentication fails	No unauthenticated update	Cross-context authentication success or delivery
Out-of-window candidate	Must not be delivered	Freshness failure or explicit policy rejection; authentication may also fail under joint reporting	No unauthenticated update	Delivery outside the acceptance window
Rollover candidate	Must remain gated before cycle confirmation	Authentication and freshness pass, followed by authenticated recovery	Only authenticated epoch update permitted	Delivery before authenticated cycle confirmation

**Table 4:** Experimental environment and principal parameters.

Item	Configuration
Target MCU	Infineon KIT_A2G_TC397_5V_TRB_S; TC397XE, TriCore CPU0 at 300 MHz
Performance environment	Target-platform timing on TC397XE; SecOC → Csm → CryIf → software Crypto Driver
Compiler	TASKING VX-toolset for TriCore v6.3r1p10, optimization level -O2
Timer	CPU0 STM0, 100 MHz, 10 ns/tick; raw latency reported
Cryptographic context	SM4-CMAC, 128-bit key; CsmJobId=17; KeyRef=0x1001; key resident in CPU0 DSPR
CAN FD parameters	500 kbit/s arbitration phase and 2 Mbit/s data phase
Baseline authenticator length	ℓ=64 bit
Freshness configuration	λ=12 bit, b=32 bit, and W=16
Cryptographic timing	Generation and verification at 8, 16, and 32 B; 30,000 samples per condition, organized as 5×6000 samples

**Table 5:** In-vehicle sensing and control messages under test.

Message	Source–Destination	Sensing or Control Semantics	Period	Deadline	Payload and Authenticated Region
PDU-A	BMS–VCU	Battery-pack total voltage, total current, and SOC status	10 ms	20 ms	16 B; bytes 0–11
PDU-B	ESC–VCU	Vehicle speed, four wheel speeds, and validity status	10 ms	20 ms	16 B; bytes 0–9
PDU-C	MCU–VCU	Motor speed, actual torque, and inverter operating status	5 ms	10 ms	16 B; bytes 0–11
PDU-D	VCU–MCU	Commanded torque, drive-enable state, and torque-limit status	5 ms	10 ms	12 B; bytes 0–7
PDU-E	BMS–VCU	Maximum/minimum cell temperature and insulation status	100 ms	200 ms	16 B; bytes 0–13

**Table 6:** Execution scale, retained semantic equivalence classes, and within-scenario variation.

Scenario	*N*_exec_	*N*_class_	Repeats/Class	Semantic Variation Retained by the Equivalence Key
Valid baseline communication	160	32	5	PDU identity and freshness-sequence position under the baseline configuration.
Payload tampering	200	40	5	PDU identity, authenticated byte/bit position, and perturbation value within the configured authenticated region.
Authenticator forgery	200	40	5	PDU identity and forged-authenticator byte/bit position or value pattern.
Historical replay and freshness rollback	220	44	5	PDU identity, historical Secured I-PDU, receiver checkpoint, and freshness distance from $FV_{\mathrm{last}}$.
Window boundary	60	12	5	Applicable PDU, configured acceptance window *W*, and candidate position relative to the window boundary.
Freshness-value rollover	220	44	5	PDU identity, pre-wrap freshness checkpoint, low-value rollover candidate, and cycle-confirmation condition.
Cryptographic-context mapping	220	44	5	Message/context pair, communication direction, security domain, and associated cryptographic-context mapping κ.
High-load injection	255	51	5	PDU identity, valid/invalid input class, background-traffic profile, phase, and message-priority relation.
Authenticator-length policy	40	8	5	Configuration identity and authenticator length ℓ∈{16,24,32,64} under the fixed attack-attempt budget and risk threshold.
Total	1575	315	5	—

**Table 7:** Message-level results by scenario.

Scenario	Valid	Invalid	IAR	FRR	DMR	IR	Principal Result
Valid baseline communication	160	0	–	0.00%	0.00%	0.00%	All valid inputs delivered on time.
Payload tampering	0	200	0.00%	–	–	0.00%	Authenticated-region modifications blocked by authentication.
Authenticator forgery	0	200	0.00%	–	–	0.00%	Forged authenticators blocked by authentication.
Historical replay and freshness rollback	0	220	0.00%	–	–	0.00%	Authentication passed, freshness failed, and all inputs were blocked.
Window boundary	0	60	0.00%	–	–	0.00%	Joint authentication/freshness rejection; no delivery.
Freshness-value rollover	0	220	0.00%	–	–	0.00%	Delivery gated and authenticated recovery triggered.
Cryptographic-context mapping	0	220	0.00%	–	–	0.00%	Cross-context inputs failed authentication.
High-load injection	126	129	0.00%	0.00%	4.76%	0.00%	All invalid inputs blocked; six valid inputs delivered late.

**Table 8:** Multidimensional response paths by test scenario.

Scenario	Valid/ Invalid	Auth. P/F	Fresh. P/F	Delivered V/I	State U/A/UA	Rejected	Auth. Resync
Freshness-value rollover	0/220	220/0	220/0	0/0	0/220/0	220	220
Historical replay and rollback	0/220	220/0	0/220	0/0	220/0/0	220	0
Valid baseline communication	160/0	160/0	160/0	160/0	0/160/0	0	0
Cryptographic-context mapping	0/220	0/220	220/0	0/0	220/0/0	220	0
Window boundary	0/60	0/60	0/60	0/0	60/0/0	60	0
Authenticator forgery	0/200	0/200	200/0	0/0	200/0/0	200	0
Payload tampering	0/200	0/200	200/0	0/0	200/0/0	200	0
High-load injection	126/129	126/129	255/0	126/0	129/126/0	129	0

Note: P/F denotes PASS/FAIL; V/I denotes valid/invalid delivery; U/A/UA denotes UNCHANGED/UPDATED_AUTH/UPDATED_UNAUTH, respectively. No ERROR or TIMEOUT event was observed in these scenarios. IAR and DMR are reported separately in Table 7.

**Table 9:** Binary-oracle ablation on the 1535 message-level execution records.

Observed Response/ Timing Signature	Count	Binary Label	CAST-SecOC Evidence	Information Lost Under Binary Scoring
Valid, on time	280	ACCEPT	Authentication PASS; freshness PASS; delivered; on time	Verification path and state-update basis are not represented.
Valid, late	6	ACCEPT	Authentication PASS; freshness PASS; delivered; LATE	Deadline violation is merged with ordinary valid delivery.
Authentication-failure block	749	REJECT	Authentication FAIL; freshness PASS; no delivery; no unauthenticated update	Authentication-based blocking is indistinguishable from other rejection paths.
Freshness-failure block	220	REJECT	Authentication PASS; freshness FAIL; no delivery; state unchanged	Replay/freshness rejection is indistinguishable from other rejection paths.
Joint authentication/ freshness rejection	60	REJECT	Authentication FAIL; freshness FAIL; no delivery	Joint rejection cannot be distinguished from a single-mechanism rejection.
Authenticated rollover-gated recovery	220	REJECT	Authentication PASS; freshness PASS; authenticated resynchronization; delivery gated	Correct authenticated recovery is indistinguishable from ordinary rejection.
Total	1535	286 ACCEPT/ 1249 REJECT	Six response/timing signatures	Reduced to two application-delivery outcomes

**Table 10:** One-sided 95% Wilson upper confidence bounds for zero-event scenarios.

Statistic	Events/Samples	Point Estimate	95% Upper Bound
Valid baseline communication, FRR	0/160	0.00%	1.66%
Direct authentication anomalies, IAR	0/400	0.00%	0.67%
Historical replay, IAR	0/220	0.00%	1.22%
Window boundary, IAR	0/60	0.00%	4.32%
Freshness-value rollover, IAR	0/220	0.00%	1.22%
Cryptographic-context mapping, IAR	0/220	0.00%	1.22%
Invalid inputs under high load, IAR	0/129	0.00%	2.05%

**Table 11:** Risk by authenticator length for q=1000 and $\theta =2^{-32}$.

Authenticator Length ℓ/bit	$P_{\mathrm{forge}}\left(1000,\ell \right)$	$P_{\mathrm{forge}}/\theta$	Policy Verdict
16	$1.5143\times 10^{-2}$	$6.50\times 10^{7}$	Non-compliant
24	$5.9603\times 10^{-5}$	$2.56\times 10^{5}$	Non-compliant
32	$2.3283\times 10^{-7}$	$1.00\times 10^{3}$	Non-compliant
64	$5.4210\times 10^{-17}$	$2.33\times 10^{-7}$	Compliant

**Table 12:** Results of the systematic configuration-mutation campaign.

Dimension	Generated	Excluded (L/E)	Executable	Killed	Survived	${\mathrm{MS}}_{d}$
*ℓ*	3	0/0	3	3	0	100.00%
λ	2	0/0	2	1	1	50.00%
*b*	2	0/0	2	1	1	50.00%
*W*	3	0/0	3	3	0	100.00%
ρ	3	1/0	2	2	0	100.00%
*A*	3	0/1	2	2	0	100.00%
κ	3	0/1	2	2	0	100.00%
$\pi^{\mathrm{timeout}}$	2	0/0	2	1	1	50.00%
$\pi^{\mathrm{persist}}$	3	1/0	2	1	1	50.00%
Total	24	2/22	20	16	4	80.00%

L/E: load-rejected/equivalent mutants.

**Table 13:** Dimensional coverage of the test set.

Coverage Dimension	Result	Covered Elements
Observable receiver conditions	Two conditions	SYNCED, ROLLOVER_GUARD
Principal freshness relations	Four relations	NEXT, STALE, OUT_OF_WINDOW, WRAP_CANDIDATE
Authenticator-length boundaries	Four values	ℓ∈{16,24,32,64} bit
Window-size boundaries	Four values	W∈{8,16,32,64}
Perturbation operators	Six operators	NONE, TAMPER_AUTH, FORGE_MAC, REPLAY, CROSS_CONTEXT, BURST_INJECTION
Message-level oracle classes	Six classes	VALID, TAMPER, REPLAY, CONTEXT, BOUNDARY_OUT, ROLLOVER
Configuration-test categories	Two categories	SYSTEMATIC_CONFIG_MUTATION, PARAMETER_POLICY
Load conditions	Two conditions	NORMAL, HIGH

**Table 14:** TC397 target-platform SM4-CMAC timing statistics.

Operation	Input/B	*N*	Mean/ms	Std./ms	Median/ms	P95/ms	P99/ms	Max/ms
Generation	8	30,000	1.5806	0.0748	1.5797	1.7011	1.7544	2.9168
Generation	16	30,000	1.9211	0.0907	1.9202	2.0682	2.1344	2.8726
Generation	32	30,000	2.5797	0.1196	2.5792	2.7758	2.8574	3.6782
Verification	8	30,000	1.6210	0.0763	1.6202	1.7448	1.8015	2.7408
Verification	16	30,000	1.9609	0.0923	1.9599	2.1109	2.1797	3.3150
Verification	32	30,000	2.6201	0.1207	2.6191	2.8198	2.9052	3.5349

**Table 15:** CAN-FD worst-case response-time results for the SecOC PDUs.

Message	Load	$T_{i}$/ms	$D_{i}$/ms	$C_{i}^{\mathrm{FD}}$/ms	$B_{i}$/ms	$Q_{i}$	$R_{i}^{\mathrm{WCRT}}$/ms	Bus-Level Residual Budget/ms
PDU-A	30%	10	20	0.220	0.300	1	2.3346	17.6654
PDU-A	85%	10	20	0.220	0.300	1	8.2596	11.7404
PDU-A	95%	10	20	0.220	0.300	1	9.2596	10.7404
PDU-B	30%	10	20	0.218	0.300	1	2.5526	17.4474
PDU-B	85%	10	20	0.218	0.300	1	8.4776	11.5224
PDU-B	95%	10	20	0.218	0.300	1	9.4776	10.5224
PDU-C	30%	5	10	0.220	0.300	1	1.9096	8.0904
PDU-C	85%	5	10	0.220	0.300	2	7.4096	2.5904
PDU-C	95%	5	10	0.220	0.300	2	8.4096	1.5904
PDU-D	30%	5	10	0.205	0.300	1	2.1146	7.8854
PDU-D	85%	5	10	0.205	0.300	2	7.8346	2.1654
PDU-D	95%	5	10	0.205	0.300	2	8.8346	1.1654
PDU-E	30%	100	200	0.224	0.300	1	2.7766	197.2234
PDU-E	85%	100	200	0.224	0.300	1	8.7016	191.2984
PDU-E	95%	100	200	0.224	0.300	1	9.7016	190.2984

## Data Availability

A public CAST-SecOC reproducibility package is available at https://github.com/a76854/CAST-SecOC (accessed on 20 September 2026). It includes the Python reference prototype, anonymized execution/configuration records, supporting metadata, and regeneration scripts for the disclosed test and mutation results. CANoe/CAPL integration materials and the external Python controller are not publicly released because they depend on the third-party-licensed Vector toolchain and contain project-specific intellectual property and configuration information. These restrictions do not affect reproduction, within the public package, of the disclosed test-vector construction, semantic-equivalence-class reduction, fixed regression set, and configuration-mutation experiments.
